# AZD1222/ChAdOx1 nCoV-19 vaccination induces a polyfunctional spike protein–specific T_H_1 response with a diverse TCR repertoire

**DOI:** 10.1126/scitranslmed.abj7211

**Published:** 2021-11-17

**Authors:** Phillip A. Swanson, Marcelino Padilla, Wesley Hoyland, Kelly McGlinchey, Paul A. Fields, Sagida Bibi, Saul N. Faust, Adrian B. McDermott, Teresa Lambe, Andrew J. Pollard, Nicholas M. Durham, Elizabeth J. Kelly, AstraZeneca/Oxford/VRC Study Group

**Affiliations:** 1 Vaccine Research Center, National Institute of Allergy and Infectious Diseases, National Institutes of Health, Bethesda, MD 20892, USA.; 2 Discovery, Research and Early Development, Oncology R&D, AstraZeneca, Gaithersburg, MD 20878, USA.; 3 Adaptive Biotechnologies, Seattle, WA 98102, USA.; 4 Oxford Vaccine Group, Department of Paediatrics, University of Oxford, and NIHR Oxford Biomedical Research Centre, Oxford OX4 6PG, UK.; 5 NIHR Southampton Clinical Research Facility and Biomedical Research Centre, University Hospital Southampton NHS Foundation Trust, and Faculty of Medicine and Institute for Life Sciences, University of Southampton, Southampton SO16 6YD, UK.; 6 The Jenner Institute, Nuffield Department of Medicine, University of Oxford, Oxford OX3 7DQ, UK.; 7 Chinese Academy of Medical Science (CAMS) Oxford Institute (COI), University of Oxford, Oxford OX3 7FZ, UK.; 8 Translational Medicine, Oncology R&D, AstraZeneca, Gaithersburg, MD 20878, USA.; 9 Translational Medicine, Microbial Sciences, Biopharmaceuticals R&D, AstraZeneca, Gaithersburg, MD 20878, USA.

## Abstract

AZD1222 (ChAdOx1 nCoV-19), a replication-deficient simian adenovirus–vectored vaccine, has demonstrated safety, efficacy, and immunogenicity against coronavirus disease 2019 in clinical trials and real-world studies. We characterized CD4^+^ and CD8^+^ T cell responses induced by AZD1222 vaccination in peripheral blood mononuclear cells from 296 unique vaccine recipients aged 18 to 85 years who enrolled in the phase 2/3 COV002 trial. Total spike protein–specific CD4^+^ T cell helper type 1 (T_H_1) and CD8^+^ T cell responses were increased in AZD1222-vaccinated adults of all ages after two doses of AZD1222. CD4^+^ T_H_2 responses after AZD1222 vaccination were not detected. Furthermore, AZD1222-specific T_H_1 and CD8^+^ T cells both displayed a high degree of polyfunctionality in all adult age groups. T cell receptor β (TCRβ) sequences from vaccinated participants mapped against TCR sequences known to react to SARS-CoV-2 revealed substantial breadth and depth across the SARS-CoV-2 spike protein for both AZD1222-induced CD4^+^ and CD8^+^ T cell responses. Overall, AZD1222 vaccination induced a polyfunctional T_H_1-dominated T cell response, with broad CD4^+^ and CD8^+^ T cell coverage across the SARS-CoV-2 spike protein.

## Interrogating T cells

The goal of the majority of coronavirus disease 2019 vaccines is to induce antibody responses against severe acute respiratory syndrome coronavirus 2. However, vaccines that can elicit T cell responses will provide another layer of protection against severe disease. Here, Swanson *et al.* evaluated T cell responses elicited by the AZD1222/ChAdOx1 nCoV-19 vaccine. The authors observed that individuals receiving two doses of the vaccine had polyfunctional CD4^+^ and CD8^+^ T cell responses specific to the vaccine-encoded spike protein. Furthermore, CD4^+^ T cell responses were primarily skewed toward T_H_1 cells. Together, these data show that the AZD1222/ChAdOx1 nCoV-19 vaccine elicits antiviral T cell responses in addition to neutralizing antibodies.

## Teaser

Polyfunctional CD4^+^ and CD8^+^ T cell responses are elicited against the SARS-CoV-2 spike protein after two doses of the AZD1222 COVID-19 vaccine.

## INTRODUCTION

The coronavirus disease 2019 (COVID-19) pandemic caused by severe acute respiratory syndrome coronavirus 2 (SARS-CoV-2) is continuing to cause substantial widespread morbidity and mortality worldwide (*1*). Cellular immunity may be critical in an individual’s response to SARS-CoV-2 infection, particularly in the face of neutralizing antibody (nAb) escape by emerging variants, and for reducing the severity of COVID-19 disease. Cases of asymptomatic COVID-19 have been associated with T cell responses without seroconversion, suggesting a role for T cell responses after SARS-CoV-2 exposure in the absence of nAbs (*2*). Furthermore, studies of patients with COVID-19 show that the presence of SARS-CoV-2–specific CD4^+^ and CD8^+^ T cells is associated with lower disease severity (*3*–*5*). SARS-CoV-2 vaccines have also been shown to elicit T cell responses (*6*–*10*), but more in-depth analyses of the functionality and breadth of vaccine-specific T cells could provide valuable information about potential determinants of protection.

AZD1222 (ChAdOx1 nCoV-19) is a replication-deficient simian adenovirus–vectored vaccine indicated for the prevention of COVID-19. The safety, efficacy, and immunogenicity of AZD1222 have been extensively demonstrated in a global clinical development program, supporting regulatory submissions for conditional or emergency use of AZD1222 (*11*–*14*). In a phase 1/2 trial conducted in the United Kingdom, COV001, AZD1222 induced marked increases in SARS-CoV-2 spike protein–specific effector T cell responses in adults aged 18 to 55 years, which peaked at day 14 after vaccination. These responses were reported as early as day 8 and were maintained through day 56, which was the latest time point analyzed (*12*). Similar responses were reported in adults aged 18 to ≥70 years in a phase 2/3 study conducted in the United Kingdom, COV002, in which SARS-CoV-2 spike protein–specific effector T cell responses peaked at day 14 after vaccination (*13*). Peak responses in adults 18 to 55 years old were characterized by interferon-γ (IFN-γ)–, interleukin-2 (IL-2)–, and tumor necrosis factor (TNF)–producing T cell helper type 1 (T_H_1) CD4^+^ T cells and CD8^+^ T cells (*11*). However, the cytokine profile of AZD1222-induced T cell responses beyond day 14 in younger adults or after a second dose of AZD1222 remains unknown. Furthermore, a full characterization of AZD1222-induced T cell cytokine responses in adults older than 55 is also undetermined (*11*).

Here, we aimed to characterize functional CD4^+^ and CD8^+^ T cell responses after the first and second doses of AZD1222 in healthy adults aged 18 to 85 years enrolled in phase 1 to 3 trials conducted in the United Kingdom. Furthermore, we describe the breadth and depth of unique SARS-CoV-2 spike protein–specific T cell responses induced by AZD1222 vaccination. These data provide a comprehensive analysis of the AZD1222 T cell response in an adult population.

## RESULTS

### Study participants

Evaluable samples were obtained from 296 participants enrolled in the COV001 and COV002 studies. Participant samples analyzed in this study were selected from three different age cohorts who received two doses of either 5 × 10^10^ virus particles of AZD1222 or control meningococcal conjugate vaccine (MenACWY) (Table 1). Of the 296 participants, 83 participants were analyzed for spike protein–specific cytokine secretion by intracellular cytokine staining (ICS), and 233 were analyzed by T cell receptor (TCR) sequencing. Twenty participants had samples in both sets of analyses.

**Table 1.** Details of participants included in this analysis.

		**Patients all ages**	**Patients aged 18** **to 55**	**Patients aged 56** **to 69**	**Patients aged ≥70**	**Patients with** **paired samples** **(day 0 and day 28** **after dose 2)**
ICS flow	MenACWY	26	9	7	10	26
	AZD1222	57	20	17	20	55
	Total	83	29	24	30	81
TCRseq	MenACWY	26	18	2	6	14
	AZD1222	207	130	26	51	114
	Total	233	148	28	57	128

### AZD1222 induced strong T_H_1-biased T cell responses to the SARS-CoV-2 spike protein

**Fig. 1.**
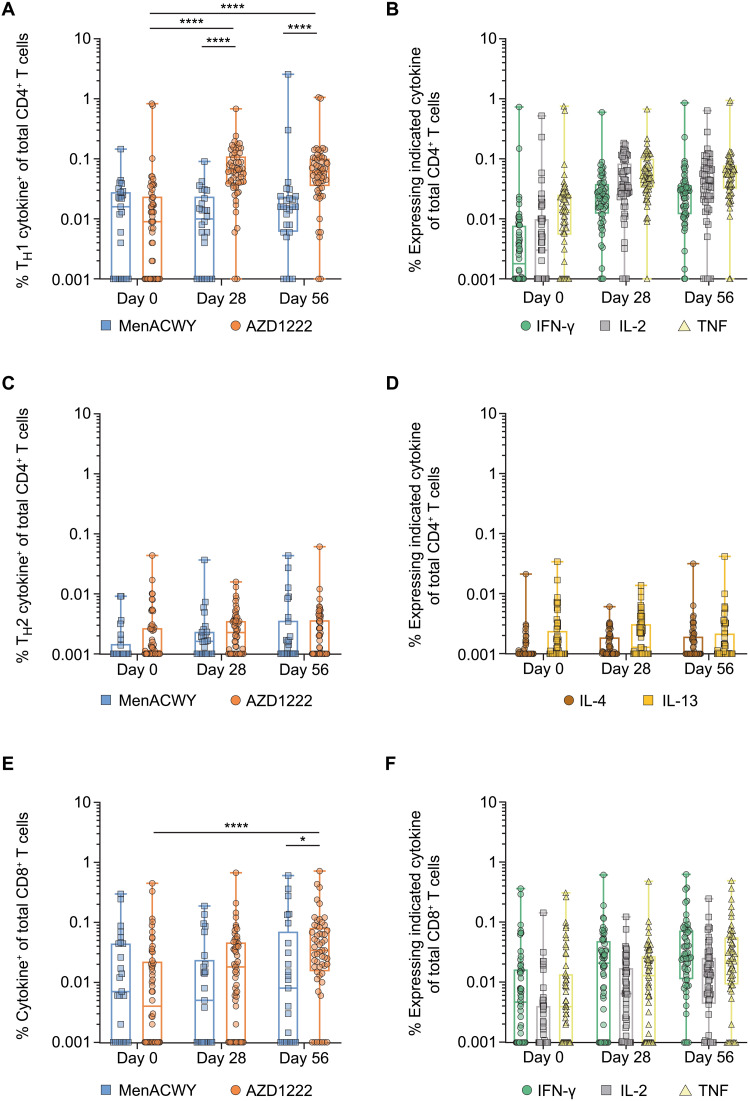
T_H_1 and CD8^+^ T cell responses are elicited after vaccination with AZD1222 or MenACWY. PBMCs from human participants vaccinated with AZD1222 or MenACWY at the indicated time point after vaccination were stimulated with SARS-CoV-2 spike peptide pools, and the intracellular cytokine response was measured. (**A**) Frequencies of total spike protein–specific CD4^+^ T cells producing any combination of T_H_1 (IFN-γ, IL-2, or TNF) cytokines are shown. (**B**) Frequencies of total spike protein–specific CD4^+^ T cells producing individual T_H_1 cytokines at the indicated time points are shown. (**C**) Frequencies of total spike protein–specific CD4^+^ T cells producing any combination of T_H_2 (IL-4 or IL-13) cytokines are shown. (**D**) Frequencies of total spike protein–specific CD4^+^ T cells producing individual T_H_2 cytokines at the indicated time points are shown. (**E**) Frequencies of total spike protein–specific CD8^+^ T cells producing any combination of IFN-γ, IL-2, or TNF after vaccination with AZD1222 or MenACWY. (**F**) Frequencies of individual CD8^+^ T cell cytokines at the indicated time points in participants vaccinated with AZD1222. For all data, responses to each peptide pool were combined to determine the total spike protein–specific response. In the box and whisker plots, the horizontal line represents median, boxes represent IQR, whiskers extend to the minimum and maximum, and symbols represent each participant. Significant differences between AZD1222 and MenACWY at each time point were determined by two-tailed Mann-Whitney tests. Significant differences between time points within each vaccine group were determined by Kruskal-Wallis test with Dunn’s test to correct for multiple comparisons. All comparisons are not significant unless stated as significant; *P < 0.05 and ****P < 0.0001.

SARS-CoV-2 spike protein–specific T cell responses from all participants vaccinated with AZD1222 or MenACWY were assessed by measuring intracellular cytokine production in peripheral blood mononuclear cells (PBMCs) after in vitro stimulation with two separate overlapping peptide pools covering the entire SARS-CoV-2 spike protein sequence. Total spike protein–specific T_H_1 responses significantly increased from a median frequency of 0.01% [interquartile range (IQR), 0.00 to 0.03] at day 0 to 0.063% (IQR, 0.04 to 0.11) in AZD1222-vaccinated participants at day 28 after vaccination and 0.062% (IQR, 0.04 to 0.10) at day 56 after vaccination (4 weeks after the second dose) (*P* < 0.0001; Fig. 1A and table S1). In MenACWY-vaccinated participants, no increases in the median frequencies of spike protein–specific T_H_1 responses were observed over time. T_H_1 responses in AZD1222-vaccinated participants were significantly greater than the median frequencies from those who received MenACWY at both day 28 (0.01% median frequency; IQR, 0.00 to 0.02; *P* < 0.0001) and day 56 (0.02% median frequency; IQR, 0.01 to 0.02; *P* < 0.0001) after vaccination although lower than the responses observed in convalescent patients with COVID-19 (0.13% median frequency; IQR, 0.12 to 0.21) (fig. S1A). The T_H_1 response followed a hierarchical pattern of cytokine production, with most spike protein–specific T_H_1 cells producing TNF (0.06% median frequency; IQR, 0.03 to 0.08), followed by IL-2 (0.04% median frequency; IQR, 0.02 to 0.07) and IFN-γ (0.03 median frequency; IQR, 0.01 to 0.04) at day 56 after vaccination (Fig. 1B and fig. S1B). Spike protein–specific T_H_2 responses were near absent after AZD1222 vaccination (Fig. 1, C and D, and fig. S1C).

In comparison with the T_H_1 response, total spike protein–specific CD8^+^ T cells were detected at lower frequencies at day 28 and day 56 after vaccination. However, at day 56 after AZD1222 vaccination, the 0.34% (IQR, 0.02 to 0.07) median frequency of spike protein–specific CD8^+^ T cells was significantly elevated compared with the 0.04% median frequency (IQR, 0.00 to 0.02; *P* < 0.0001) at day 0 and compared with the median frequency of 0.01% (IQR, 0.00 to 0.06; *P* = 0.0296) at day 56 in participants vaccinated with MenACWY (Fig. 1E and table S1). Spike protein–specific CD8^+^ T cell responses induced by AZD1222 were also equivalent to those observed in convalescent patients with COVID-19 (0.04% median frequency; IQR, 0.02 to 0.10) (fig. S2A). In contrast to the T_H_1 response, at day 56, most spike protein–specific CD8^+^ T cells produced IFN-γ with a median frequency of 0.03% (IQR, 0.01 to 0.07), followed by TNF (0.02% median frequency; IQR, 0.01 to 0.05) and IL-2 (0.01% median frequency; IQR, 0.00 to 0.03) (Fig. 1F and fig. S2B). Using a twofold increase in any spike protein–specific CD4^+^ and CD8^+^ T cell responses, study participants vaccinated with AZD1222 induced response rates of 79.2 and 68.6%, respectively, 28 days after a second dose (table S2). These data demonstrate that AZD1222 elicited a robust T_H_1-dominated T cell response with an expanded CD8^+^ T cell response after vaccination compared with day 0 albeit at a smaller magnitude than the CD4^+^ response.

### AZD1222 vaccination generated a polyfunctional CD4^+^ T cell cytokine response across adult age groups

**Fig. 2.**
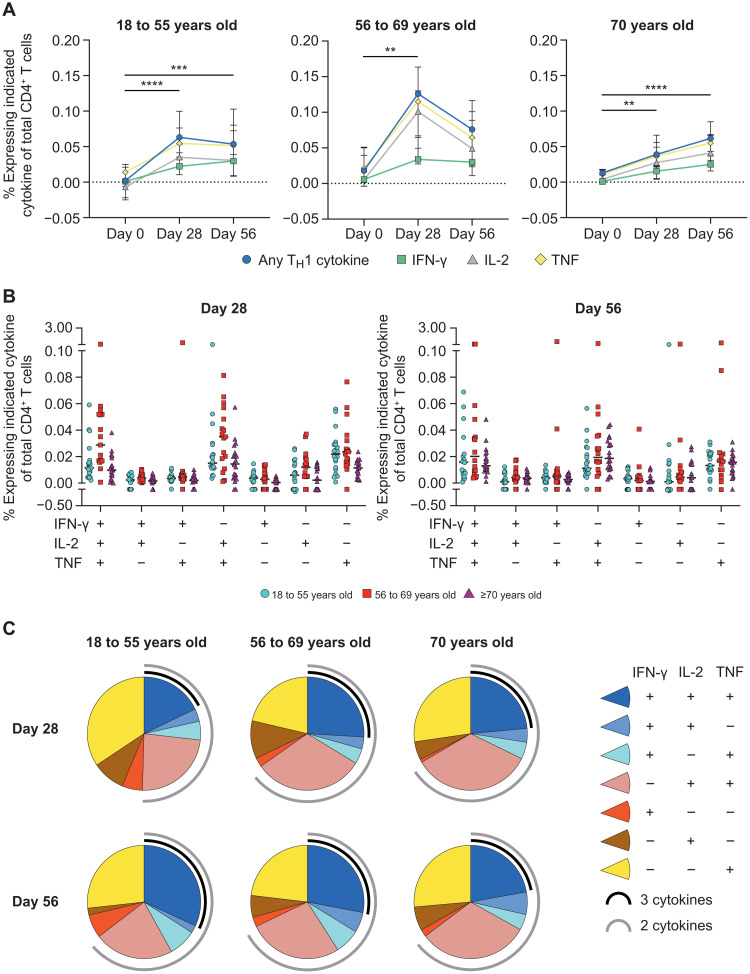
Age-specific CD4^+^ T cell responses are observed after AZD1222 vaccination. (**A**) Median frequencies with IQRs are shown for CD4^+^ T cells from participants within each age cohort producing IFN-γ, IL-2, TNF, or any combination of these cytokines at the indicated time points after stimulation with SARS-CoV-2 spike peptide pools. Significant differences between time points within each vaccine group were determined by Kruskal-Wallis tests with Dunn’s test to correct for multiple comparisons. All comparisons are not significant unless stated as significant; **P < 0.01, ***P < 0.001, and ****P < 0.0001. (**B**) Frequencies of antigen-stimulated CD4^+^ T cells producing each combination of IFN-γ, IL-2, and TNF cytokines at day 28 (left) or day 56 (right) after vaccination are shown. Individual participant responses are shown with median represented by the horizontal line. (**C**) Pie graphs indicate the total proportion of spike protein–specific T_H_1 cytokine production averaged for all participants within the indicated age groups at day 28 and day 56 after vaccination. A proportion of multicytokine responses are represented by the black (three cytokines) and gray (two cytokines) arcs.

To determine whether CD4^+^ T cell responses to AZD1222 vaccination varied by age, we performed subgroup analyses according to participants’ age (18 to 55, 56 to 69, and ≥70 years). Significant increases in spike protein–specific CD4^+^ T cell responses at day 28 over day 0 were observed for participants within each age group (18 to 55, *P* < 0.0001; 56 to 69, *P* = 0.0015; ≥70 years, *P* = 0.0024; Fig. 2A). The median frequency of spike protein–specific CD4^+^ T cells producing any T_H_1 cytokine increased from 0.00% (IQR, 0.00 to 0.02) at day 0 to 0.05% (IQR, 0.03 to 0.10) at day 56 for 18- to 55-year-olds, 0.02% (IQR, 0.01 to 0.05) at day 0 to 0.08% (IQR, 0.04 to 0.11) at day 56 for 56- to 69-year-olds, and 0.01% (IQR, 0.00 to 0.02) at day 0 to 0.06% (IQR, 0.04 to 0.08) at day 56 in ≥70-year-olds. Individual T_H_1 cytokine production followed a similar hierarchical pattern within each age group (TNF > IL-2 > IFN-γ) (Fig. 2A, fig. S3A, and table S3). However, the kinetics of the AZD1222-specific T_H_1 cell response differed by age. In participants aged 18 to 55 years, the median frequency of spike protein–specific CD4^+^ T cells producing any T_H_1 cytokine increased to 0.06% (IQR, 0.04 to 0.09) at day 28 and remained at a consistent frequency through day 56 after vaccination (Fig. 2A). In participants aged 55 to 69 years, any response median frequency of the spike protein–specific T_H_1 peaked at 0.13% (IQR, 0.07 to 0.16) on day 28 and declined by day 56 despite receiving a second vaccine dose. Although T_H_1 cell responses observed at day 28 after vaccination were at a lower frequency in participants aged ≥70 years compared with other age groups (0.04% median frequency; IQR, 0.02 to 0.06), spike protein–specific responses demonstrated an increased frequency at day 56, potentially aided by the second vaccine dose at day 28. No age-specific differences in AZD1222-specific T_H_1 cell responses were observed at day 56.

The frequencies (Fig. 2B) and proportions (Fig. 2C) of AZD1222-specific T_H_1 cells producing each combination of T_H_1 cytokines were compared among age groups at day 28 and day 56 after vaccination. Spike protein–specific T_H_1 cells displayed a high degree of polyfunctionality at day 28 after vaccination in all three age groups, with 50% of the spike protein–specific CD4^+^ T cells in 18- to 55-year-olds and 65% of the responses in 56- to 69- and ≥70-year-olds producing two or more T_H_1 cytokines. Spike protein–specific T_H_1 cells remained polyfunctional through day 56 among participants aged 56 to 69 and ≥70 years. However, in participants aged 18 to 55 years, the proportion of polyfunctional spike protein–specific T_H_1 cells increased to 64%. Furthermore, at day 56, spike protein–specific T_H_1 cells producing all three cytokines represented 32% of the response in participants aged 18 to 55, 28% in participants aged 56 to 69 years, and 23% in participants aged ≥70 years. These data show that AZD1222 induces a polyfunctional T_H_1 cell response that is equivalent in frequency and functionality across all adult age groups at day 56 after vaccination.

### Polyfunctional CD8^+^ T cell responses are generated after AZD1222 vaccination

**Fig. 3.**
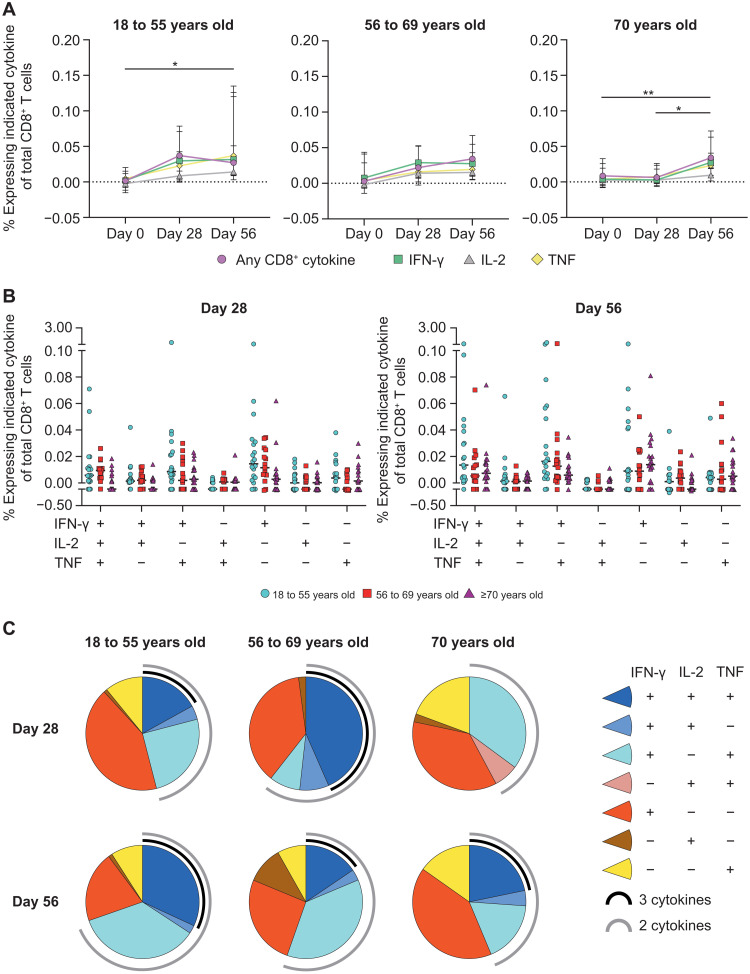
Age-specific CD8^+^ T cell responses are elicited after AZD1222 vaccination. (**A**) Median frequencies with IQRs of CD8^+^ T cells producing IFN-γ, IL-2, TNF, or any combination of these cytokines are shown for the indicated time points after stimulation with SARS-CoV-2 spike peptide pools. Significant differences between time points within each vaccine group were determined by Kruskal-Wallis tests with Dunn’s test to correct for multiple comparisons. All comparisons are not significant unless stated as significant; *P < 0.05 and **P < 0.01. (**B**) Frequencies of antigen-stimulated CD8^+^ T cells producing each combination of IFN-γ, IL-2, and TNF cytokines at day 28 (left) or day 56 (right) after vaccination at the individual level are shown. Individual participant responses are shown with the median represented by the horizontal line. (**C**) Pie graphs indicate the total proportion of spike protein–specific CD8^+^ T cell cytokine production within the indicated age groups at day 28 and day 56 after vaccination. A proportion of multicytokine responses are represented by the black (three cytokines) and gray (two cytokines) arcs.

AZD1222-specific CD8^+^ T cell responses were generated in all age groups but at lower frequencies compared with the T_H_1 responses. Spike protein–specific CD8^+^ T cell responses peaked at day 28 after vaccination in participants aged 18 to 55 years (0.04% median frequency; IQR, 0.00 to 0.07) and 56 to 69 years (0.02% median frequency; IQR, 0.01 to 0.05), with little change at day 56 for 18- to 55-year-olds (0.03% median frequency; IQR, 0.01 to 0.11) or 56- to 69-year-olds (0.03% median frequency; IQR, 0.02 to 0.07) (Fig. 3A, fig. S3B, and table S3). However, participants aged ≥70 years seemed to benefit from a second vaccine dose, because significant expansion of spike protein–specific CD8^+^ T cells was observed at day 56 after vaccination (0.03% median frequency; IQR, 0.02 to 0.06) above day 0 (0.01% median frequency; IQR, 0.00 to 0.03; *P* = 0.0103) and day 28 (0.01% median frequency; IQR, 0.00 to 0.02; *P* = 0.0050). By day 56, no statistical differences in spike protein–specific CD8^+^ T cell responses were observed among age groups.

The frequency and proportion of each combination of spike protein–specific CD8^+^ T cell cytokines were also examined for each age group. At day 28 after vaccination, 51% of spike protein–specific CD8^+^ T cells in participants aged 18 to 55 and 58% of spike protein–specific CD8 T cells in participants aged ≥70 years only produced a single cytokine (Fig. 3, B and C). However, participants aged 56 to 69 years generated a multicytokine response at day 28 after vaccination, with 72% of spike protein–specific CD8^+^ T cells producing two or more cytokines. Although the total frequency of spike protein–specific CD8^+^ T cells in participants aged 18 to 55 years did not increase from day 28 to day 56, the proportion of multicytokine-producing cells increased to 74% of all responding CD8^+^ T cells, with 32% of responding CD8^+^ T cells producing TNF, IL-2, and IFN-γ. Frequencies of polyfunctional spike protein–specific CD8^+^ T cells in participants aged ≥70 years also increased from day 28 to day 56, with 23% producing TNF, IL-2, and IFN-γ at day 56. Together, these data demonstrate that AZD1222-specific CD8^+^ T cells are largely polyfunctional and that the size of the response is equivalent across all age groups at day 56 after vaccination. The correlation between the magnitude of nAb response and frequency of spike protein–specific CD4^+^ T_H_1 T cell response was further examined in a subset of AZD1222- and MenACWY-vaccinated study participants (fig. S4). Although no correlations were observed between the magnitude of nAb responses and frequency of T_H_1 responses after a first or second dose of AZD1222, this may be influenced by the limited sample size (*n* = 34) for which both humoral and cell-mediated responses were available. If these data are observed in a larger cohort, then it may indicate that T cell responses can complement a lower-magnitude nAb response in those vaccinated with AZD1222.

### AZD1222-specific T cell responses were genetically diverse and covered a broad range of SARS-CoV-2 spike protein epitopes

To better understand the diversity and specificity of the T cell response to SARS-CoV-2 spike protein after AZD1222 vaccination, we performed TCRβ chain sequencing on 233 PBMC samples collected at day 0 and day 28 after the second dose. Individual TCRs in each repertoire were compared against a library of TCRs with known specificity for SARS-CoV-2 that had been generated using the Multiplex Identification of T cell Receptor Antigen Specificity (MIRA) platform (*15*). The total number of unique and total antigen-specific TCRs before and after vaccination was characterized for each repertoire. PBMCs from study participants treated with two doses of AZD1222 at different dose intervals were used to increase the power of the TCR repertoire analysis. In addition to the participants used in the ICS analysis who received a second dose at about 4 weeks (18 to 60 days) after initial vaccination, a second cohort of participants who received a second dose at about 12 weeks (61 to 130 days) after vaccination was also analyzed. Participants vaccinated with AZD1222 at about the 4-week and 12-week second dose schedules both had a significant increase in the fraction of total T cells that were spike protein specific (SARS-CoV-2–associated T cells of total T cells; depth) (*P* < 0.0001; fig. S5A) and in the fraction of unique TCRs that were spike protein specific (SARS-CoV-2–associated unique TCRs of total unique TCRs; breadth) (fig. S5B) after the second dose. There was no statistical difference in either the depth or the breadth of the responses between the two dosing regimens. Because of this equivalency, data from these two dosing regimens were combined into single “day 0” and “day 28 after the second dose” groups.

**Fig. 4.**
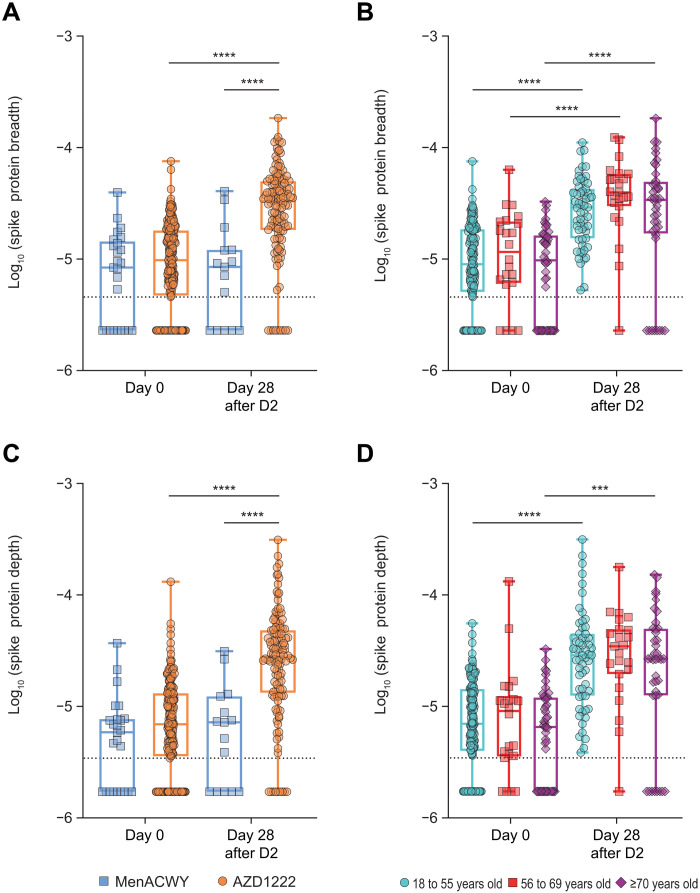
Spike protein–specific TCR breadth and depth increased after vaccination with two doses of AZD1222. (**A**) Spike protein–specific TCR breadth after dose two (D2) of vaccination with MenACWY (blue) or AZD1222 (orange) is shown. Breadth indicates SARS-CoV-2–associated unique TCRs of total unique TCRs. (**B**) Spike protein–specific TCR breadth is shown disaggregated by age. (**C**) Spike protein–specific TCR depth after vaccination with MenACWY (blue) or AZD1222 (orange) is shown. Depth indicates SARS-CoV-2–associated T cells of total T cells. (**D**) Spike protein–specific TCR depth is shown disaggregated by age. Data are log_10_-transformed, where all 0 values have been converted to half of the lowest nonzero value. In the box and whisker plots, the horizontal line represents median, boxes represent IQR, whiskers extend to the minimum and maximum, and symbols represent each participant. The dotted lines represent the lower detected value. Significant differences were determined by one-way ANOVA followed by Sidak’s multiple comparisons tests. All comparisons are not significant unless stated as significant; ***P < 0.001 and ****P < 0.0001.

Looking first at the breadth of spike protein–specific TCRs from AZD1222-vaccinated participants, a significant increase was observed from day 0 to day 28 after the second dose (*P* < 0.0001; Fig. 4A). Furthermore, spike protein–specific TCR breadth was also significantly elevated in AZD1222-vaccinated participants compared with MenACWY-vaccinated participants at day 28 after the second dose (*P* < 0.0001; Fig. 4A). No increase in TCR breadth of non–spike protein–specific SARS-CoV-2 TCRs was observed in either AZD1222- or MenACWY-vaccinated participants (fig. S6A). When participants were separated by age cohorts, all three age groups had a significant increase in spike protein–specific TCR breadth from day 0 to day 28 after the second dose (*P* < 0.0001; Fig. 4B). In addition to TCR breadth, total TCR templates were also quantified for each group. Further confirming the ICS data (Fig. 1), the number of spike protein–specific TCRs, or depth, was significantly increased in AZD1222-vaccinated participants at day 28 after the second dose compared with day 0 and also compared with MenACWY-vaccinated participants (*P* < 0.0001; Fig. 4C). The trends observed with regard to spike protein–specific TCR depth after vaccination were the same across age groups, but in the 56 to 69 years group, these responses did not meet significance for depth of response, likely due to the lower power given the smaller number of analysis points for this age group (*P* = 0.14). Participants aged 18 to 55 years and ≥70 years displayed significant increases in spike protein–specific TCR depth after vaccination (*P* < 0.0001 and *P* = 0.0003, respectively; Fig. 4D). In addition, two convalescent patients with COVID-19 were sequenced, and the depth and breadth of spike protein–specific T cells were comparable to patients at day 28 after the second dose (table S4). There were no changes in depth within non–spike protein–specific TCRs in AZD1222- or MenACWY-vaccinated participants (fig. S6B).

**Fig. 5.**
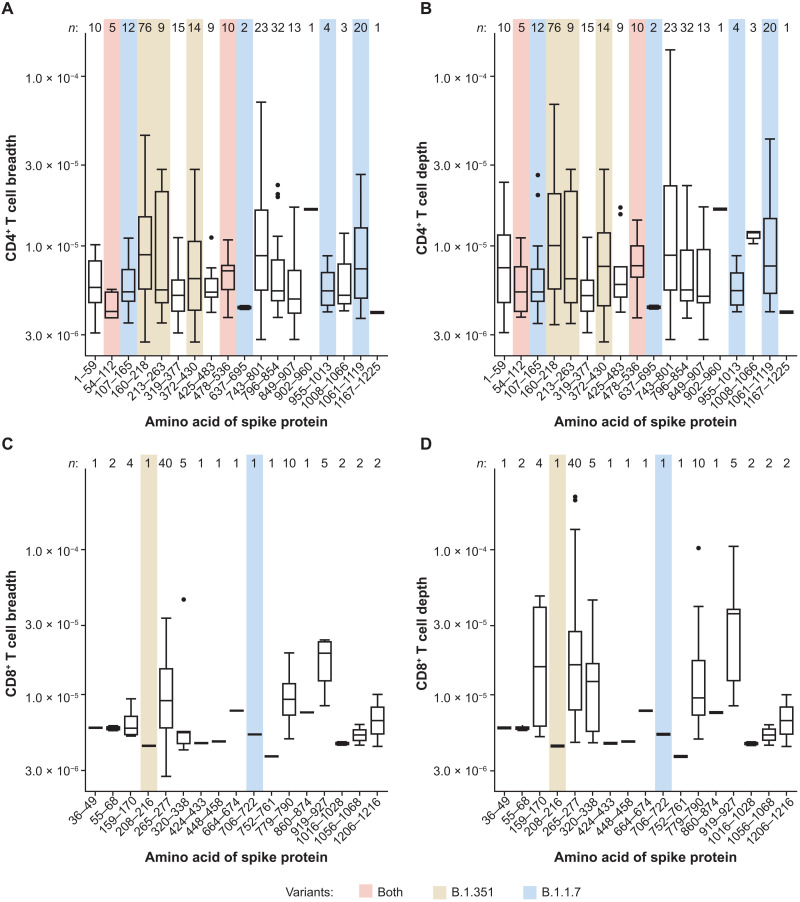
Spike protein–specific CD4^+^ and CD8^+^ T cell responses demonstrate substantial breadth and depth across the entire spike protein. TCR sequences from participants vaccinated with AZD1222 at day 28 after the second dose were mapped against TCR sequences known to react to SARS-CoV-2. (**A** and **B**) CD4^+^ T cell breadth (A) and depth (B) were analyzed for each participant. (**C** and **D**) CD8^+^ T cell breadth (C) and depth (D) were analyzed for each participant. The number of participants displaying responses for each epitope region is indicated at the top of each plot (*n*). Sequences of known variants were aligned to known Multiplex Identification of T cell Receptor Antigen Specificity (MIRA) antigen locations, with any mutations observed in B.1.351 (yellow), B.1.1.7 (blue), or both (red) highlighted. In the box and whisker plots, the horizontal line represents median, boxes represent IQR, whiskers extend to the 5th and 95th percentiles, and individual symbols represent outlier samples.

To better understand the breadth and depth of the T cell response in AZD1222-vaccinated participants, we mapped each unique spike protein–specific TCR sequence to the region of the spike protein to which it reacted in the MIRA data (*15*). Spike protein–specific CD4^+^ T cell responses spanned the entire spike protein (Fig. 5, A and B, and table S5). Dominant responses (those with the highest frequency) were observed in the N-terminal region 160–218 and the C-terminal side 743–854. Fewer unique spike protein–specific CD8^+^ T cell TCRs were sequenced, likely due to the lower response after vaccination at these time points (Fig. 1) and the greater human leukocyte antigen (HLA) restriction of class I peptides. Half of all unique CD8^+^ TCRs mapped to a single region of the spike protein, with the other TCRs spread evenly across the other regions (Fig. 5, C and D). Overlaying known mutations in variants of concern, including B.1.1.7 and B.1.351, indicates that although nearly 30% of unique TCRs mapped to a single region of the spike protein that is mutated in the B.1.351 SARS-CoV-2 variant, nearly half of all unique spike protein–specific TCRs recognized epitopes outside of the mutated regions found in the B.1.351 and B.1.1.7 variants. Moreover, the mutation within this single region is a single point mutation, D215G. Therefore, these data demonstrate that AZD1222 vaccination induces an expansion of SARS-CoV-2 spike protein–specific T cells with TCR sequences that map to a number of epitopes across the entire spike protein.

## DISCUSSION

Correlates of protection against COVID-19 are currently undetermined (*16*); however, cellular immunity is associated with positive outcomes (*3*–*5*). Here, we have provided a comprehensive assessment of the T cell response to AZD1222 vaccination. AZD1222 vaccination elicited a robust and polyfunctional T_H_1-dominated T cell response to the SARS-CoV-2 spike protein with a spike protein–specific CD8^+^ T cell response across all age groups 28 days after the second dose. These responses may be critical in reducing the clinical symptoms associated with COVID-19 disease, as evidenced by longitudinal follow-up of patients with SARS-CoV-2 infection, where rapid CD4^+^ T cell responses in acute COVID-19 were shown to be associated with mild disease and accelerated viral clearance, and early appearance of SARS-CoV-2–specific T cells was associated with shorter duration of infection (*17*). In a study that measured SARS-CoV-2–specific antibodies, CD4^+^, and CD8^+^ T cells in participants with a range of COVID-19 disease severities, severe or fatal disease was shown to be associated with minimal or lacking SARS-CoV-2–specific CD4^+^ and CD8^+^ T cell responses (*3*). Furthermore, SARS-CoV-2–specific CD4^+^ T cells were associated with less severe disease. SARS-CoV-2–specific CD8^+^ T cells were also associated with lower disease severity, reinforcing the potential importance of T cell–mediated immunity for minimizing disease caused by SARS-CoV-2 infection. Last, there is evidence with some vaccinations, such as influenza, that T cell–mediated immunity may be a more reliable correlate of protection than antibody responses in older adults (*18*). The AZD1222-primed T cell responses that formed across all age groups may provide critical protection from severe illness due to COVID-19, as seen in vaccinated participants in these trials.

Strong T_H_1 responses are an important safety indicator for vaccines against respiratory pathogens. Previous whole inactivated virus vaccines for measles (*19*) and respiratory syncytial virus (*20*) led to vaccine-associated enhanced respiratory disease. This disease, characterized by an intense allergic inflammation of the airways, is driven by vaccine-specific T_H_2 cells (*21*). Furthermore, vaccination of mice with SARS-CoV-1 and Middle East respiratory syndrome coronavirus vaccines has been shown to result in immunopathology that was attributed to T_H_2-biased responses (*22*, *23*). T_H_1-predominant responses and balanced CD4^+^ and CD8^+^ T cell responses are less likely to induce immunopathology and are therefore preferred COVID-19 vaccine characteristics. Spike protein–specific T_H_2 responses were minimal after AZD1222 vaccination in all age groups. These data are consistent with other SARS-CoV-2 vaccines, in which similar T_H_1-dominated responses have been reported (*6*–*9*).

When we further characterized this T cell response, most spike protein–specific T_H_1 and CD8^+^ T cells were polyfunctional across all age groups. Whereas previous data have suggested that T cells, as analyzed by IFN-γ enzyme-linked immunosorbent spot, do not increase after a second dose of AZD1222, the increases in polyfunctionality that are seen after the second dose suggest that the quality, rather than quantity, of the response may be improved by a two-dose regimen. The quality of the T cell response by cytokine characterization can give clues as to how well T cells are performing effector functions and organizing immune responses (*24*). Numerous studies have demonstrated that both vaccine- and infection-elicited, multicytokine-producing T cells correlate with enhanced protection against cytomegalovirus (CMV), hepatitis C virus, human herpesvirus-8, HIV, influenza virus, yellow fever virus, and *Leishmania major* (*25*–*30*). The polyfunctional responses that we observe in our analyses therefore support the contribution of T cell immunity to the protective effect of AZD1222 vaccination. Although there was more limited polyfunctionality of CD8^+^ T cell responses after the first dose of AZD1222 in adults aged ≥70 years, after a second dose, CD8^+^ T cells improved in quantity and polyfunctionality. Therefore, two doses of AZD1222 may be required in adults aged ≥70 years to achieve the same degree of polyfunctionality that is achieved in other age groups after the first dose. Data from clinical trials and emerging real-world evidence in individuals aged ≥65 have demonstrated the effectiveness of AZD1222 for the protection of COVID-19 disease after one or two doses (*13*, *31*–*33*).

Although T cells are important for minimizing disease caused by SARS-CoV-2, nAbs have also been found to be associated with protection against reinfection (*34*). B cells depend on CD4^+^ T cell help for the development of pathogen-specific antibody responses (*35*). Anti–spike protein receptor binding domain antibody responses in patients who have recovered from COVID-19 correlated with the magnitude of spike protein–specific CD4^+^ T cell responses (*36*). Such antibody responses are facilitated specifically by a subset of CD4^+^ T cells called T follicular helper (Tfh) cells through the development of germinal centers in secondary lymphoid tissues (*37*). Circulating Tfh cells have been detected in patients who have recovered from COVID-19 (*38*) and have even been shown to be prominent among specific CD4^+^ T cells in individuals during acute COVID-19 or after convalescence (*3*). Characterizing the frequency and phenotype of circulating Tfh cells after AZD1222 vaccination may be informative to future studies.

In the present study, AZD1222-expanded T cells were reactive to a variety of epitopes spanning the length of spike protein. Analysis by TCR sequencing (TCRseq) showed an increase in both breadth and depth across multiple epitopes of the SARS-CoV-2 spike protein. Increased T cell breadth is an important protective mechanism against viruses that genetically diverge from parental strains (*39*–*42*). Recently, the SARS-CoV-2 spike genome has accumulated mutations, resulting in the emergence of SARS-CoV-2 variants, including the B.1.351 lineage that was first identified in South Africa and the B.1.1.7 lineage that was first identified in the United Kingdom (*43*, *44*). Efficacy of AZD1222 against SARS-CoV-2 variants B.1.351 and B.1.1.7 investigated in Syrian hamsters showed that, despite an observed reduction in nAb titers against B.1.351 in AZD1222-vaccinated hamsters compared with those against B.1.1.7, there was evidence of protection in the lower respiratory tract against both variants after AZD1222 vaccination (*45*). AZD1222 vaccination has been shown to provide protection against symptomatic disease in adults aged ≥18 years caused by the B.1.1.7 lineage, shortening the duration of shedding and viral load, which may result in reduced transmission of disease (*46*). Although AZD1222 was not able to prevent mild to moderate symptomatic infection against B.1.351 in humans, we believe that the vaccine may still play a protective role against severe disease, and further study is warranted. Recent studies in patients exposed to SARS-CoV-2 demonstrated that CD4^+^ and CD8^+^ T cell responses were not affected by mutations found in the B.1.351 and B.1.1.7 variants (*47*, *48*). The breadth of T cell responses across multiple viral epitopes spanning the length of SARS-CoV-2 spike protein after vaccination with AZD1222 is similar to the response after Ad26.CoV2.S vaccination (*49*) and suggests that the cellular immune response to AZD1222 may be resilient to point mutations in SARS-CoV-2.

Limitations of the study included that it was run in the United Kingdom, with a majority of participants who were white and of British descent and with more females than males in the overall population. This may have therefore resulted in underestimating clonal diversity induced by AZD1222, due to a population with a more limited HLA profile and may be a factor responsible for reduced spike protein–specific CD8^+^ T cell clonal diversity and lower frequencies compared with corresponding T_H_1 responses. Furthermore, spike protein–specific CD8^+^ T cell frequencies may have been reduced because samples were analyzed at day 28 after vaccination, which is 2 weeks past the peak time point of the T cell responses (*13*), although they were still comparable to CD8^+^ T cell responses from convalescent patients with COVID-19. Lack of availability of biospecimens at the day 14 time point in the present study prevented analyses of AZD1222-induced T cell responses against SARS-CoV-2 and characterization of the TCR repertoire at this peak time point. Furthermore, in this study, only six participants vaccinated with AZD1222 were seropositive at day 0, providing limited data for the effect of AZD1222 vaccination on seropositive participants. Despite these study limitations, our observations are still consistent with data that SARS-CoV-2–specific CD4^+^ T cell responses are more prominent than CD8^+^ T cell responses after infection (*36*) and vaccination (*7*, *8*, *10*).

In summary, a combination of antibody and T cell immunity is likely needed to provide long-term protection against SARS-CoV-2 infection (*50*). Previous studies have shown that AZD1222 leads to a robust nAb response across multiple age groups (*12*, *13*). Here, we demonstrate that AZD1222 vaccination also induces a polyfunctional T_H_1-dominated T cell response to SARS-CoV-2 spike protein in all adult age groups, including expansion of SARS-CoV-2 spike protein–specific CD4^+^ and CD8^+^ T cells, with unique TCR sequences that mapped to multiple epitope regions across the entire spike protein, which may provide long-lasting protection from severe disease associated with SARS-CoV-2 variants.

## MATERIALS AND METHODS

### Study design

Healthy adults aged 18 to ≥70 years were enrolled in a single-blind, randomized, and controlled phase 2/3 trial for the SARS-CoV-2 vaccine, AZD1222 (ChAdOx1 nCoV-19), as described in the previously published safety and immunogenicity report (*13*). Full descriptions of the methods of the studies have been previously published, including full study protocols (*12*, *13*). Briefly, participants were enrolled in an age-escalation manner, into 18 to 55, 56 to 69, and ≥70 years immunogenicity subgroups. Participants were randomly assigned to receive either AZD1222 or control MenACWY, using block randomization. Because of a dosing error in the study, some participants received a low dose (2.2 × 10^10^ virus particles) rather than the intended standard dose (5 × 10^10^ virus particles) as their first dose. A licensed vaccine comparator such as MenACWY has the potential to provide a benefit to participants (prevention of infection with *Neisseria meningitidis*) and facilitates the participant blinding, who, upon control vaccination, may experience local and general adverse events similar to those experienced by those vaccinated with the experimental vaccine. Participants aged 18 to 55 years were randomly assigned (1:1) to either two doses of AZD1222 or two doses of MenACWY. Participants aged 56 to 69 years were randomly assigned (3:1:3:1) to one dose of AZD1222, one dose of MenACWY, two doses of AZD1222, or two doses of MenACWY. Participants aged ≥70 years were randomly assigned (5:1:5:1) to one dose of AZD1222, one dose of MenACWY, two doses of AZD1222, or two doses of MenACWY. Prime-booster regimens were given 28 days apart. Participants were then recruited to the standard-dose cohort (5 × 10^10^ virus particles of AZD1222), and the same randomization procedures were followed, except participants aged 18 to 55 years were randomly assigned in a 5:1 ratio to two doses of AZD1222 or two doses of MenACWY. Participants, clinical investigators, and the laboratory team, but not the staff administering the vaccine, were masked to vaccine allocation for the duration of the study.

For the analysis performed in this study, participant samples, for whom PBMCs were available at matched time points, were selected from all three age cohorts who received either two doses of AZD1222 (5 × 10^10^ virus particles) or control MenACWY (Table 1). In total, samples were obtained from 296 unique participants in studies COV001 and COV002. A windowing convention was applied to ICS and TCRseq data. For ICS, day 28 refers to 28 ± 7 days after the first dose, and day 56 refers to 28 ± 7 days from the second dose (or about 56 days after the first dose). Written informed consent was obtained from all participants, and the trials were performed in accordance with the principles of the Declaration of Helsinki and Good Clinical Practice. COV001 was approved in the United Kingdom by the Medicines and Healthcare products Regulatory Agency (reference 21584/0424/001-0001) and the South-Central Berkshire Research Ethics Committee (reference 20/SC/0145). COV002 was sponsored by the University of Oxford and approved in the United Kingdom by the Medicines and Healthcare products Regulatory Agency (reference 21584/0428/001-0001) and the South-Central Berkshire Research Ethics Committee (reference 20/SC/0179). Vaccine use was authorized by Genetically Modified Organisms Safety Committees at each participating site.

Samples from 83 participants were used for ICS analysis and samples from 233 participants for TCR analysis, with 20 participant samples present in both datasets (Table 1). One patient in TCR analysis from the 18 to 55 age group was confirmed to have received a lower first dose (2.25 × 10^10^ virus particles) and a standard second dose (5 × 10^10^ virus particles) of AZD1222.

### ICS assay and analysis

PBMCs were isolated from vaccine recipient blood samples within 6 hours of venipuncture and cryopreserved at below −150°C before use in ICS assays. Human convalescent samples were obtained after symptom onset from adults with previous SARS-CoV-2 infection. Specimens were collected after participants provided written informed consent under an institutional review board–approved protocol at the National Institutes of Health Clinical Center (NCT00067054). PBMCs were isolated and cryopreserved similarly to those from vaccine recipients. ICS was performed on samples obtained at day 0, day 28, second dose (if different from day 28), and day 28 after the second dose.

Antibody titration of all antibodies included in the analysis was performed before the analysis of clinical specimens. The ICS assay was used to evaluate T cell responses, as previously described (*10*). Briefly, PBMCs were thawed and rested overnight before being stimulated with pools of 15-mer peptides overlapping by 10 amino acids covering the N terminus of SARS-CoV-2 spike protein up to the furin cleavage site (S1 pool) and the C terminus of the SARS-CoV-2 spike protein up to the furin cleavage site (S2 pool) for 6 hours at 37°C with 5% CO_2_. Peptide pools were custom-ordered from JPT Peptide Technologies GmbH and were >85% pure.

After stimulation, cells were stained and analyzed as described previously (*51*). Briefly, cells were washed with phosphate-buffered saline (PBS) and stained with viability dye for 20 min at room temperature, followed by surface stain for 20 min at room temperature, cell fixation, and permeabilization with a BD Cytofix/Cytoperm kit (catalog no. 554714) for 20 min at room temperature and then intracellular stain for 20 min at room temperature. All antibody staining was performed in the dark. Surface staining and all pre-fixation washes were performed with fluorescence-activated cell sorting buffer (PBS + 1% fetal bovine serum + 0.02% NaN_3_), and intracellular staining and postfixation washes were performed in 1× BD Perm/Wash buffer (BD Biosciences, catalog no. 554714). See table S6 for a complete list of antibodies used. Upon completion of staining, cells were collected on a BD FACSymphony flow cytometer. Samples were invalidated if <20,000 live CD3^+^ T cells were collected. On average, ≥280,000 cells were collected for each sample. Samples were analyzed using FlowJo 10.6.2 (BD Biosciences). Anomalous “bad” events were separated from “good” events using FlowAI (*52*). “Good events” were used for all downstream gating. Gating strategy can be found in fig. S7. Individual cytokines were plotted on the *y* axis versus CD69^+^ cells on the *x* axis, and only CD69^+^ events were used to determine positive responses. No cytokine-positive responses were detected above background in the CD69^−^ gate. Representative flow plots for cytokine gating are shown in fig. S8. CD69^+^ activated cells were increased over baseline after AZD1222 vaccination but did not differ between peptide pools or by age groups (fig. S9). All antigen-specific cytokine frequencies are reported after background subtraction of identical gates from the same sample incubated with negative control stimulation with dimethyl sulfoxide.

Assay qualification was performed by the Vaccine Immunology Program at the Vaccine Research Center, National Institute of Allergy and Infectious Diseases, National Institutes of Health. Assay qualification included assessment of T_H_1 and T_H_2 specificity, T_H_1 and T_H_2 intra-assay precision, inter-assay precision, and SARS-CoV-2 peptide pool validation. T_H_1 and T_H_2 specificity was conducted using PBMCs from acutely infected CMV donors (for T_H_1 specificity) or *Filaria* parasite donors (for T_H_2 specificity), given the established cytokine profiles of these pathogens.

### TCRβ chain sequencing

Immunosequencing of the complementarity-determining region 3 (CDR3) regions of human TCRβ chains was performed using the ImmunoSEQ Assay (Adaptive Biotechnologies). Extracted genomic DNA was amplified in a bias-controlled multiplex polymerase chain reaction (PCR), followed by high-throughput sequencing. Sequences were collapsed and filtered to identify and quantitate the absolute abundance of each unique TCRβ CDR3 region for further analysis as previously described (*53*–*55*). The fraction of T cells was calculated by normalizing TCRβ template counts to the total amount of DNA usable for TCRseq, where the amount of usable DNA was determined by PCR amplification and sequencing of several reference genes that are expected to be present in all nucleated cells.

### Mapping of SARS-CoV-2 TCRβ sequences

TCR sequences from participants receiving AZD1222 were mapped against a set of TCR sequences that are known to react to SARS-CoV-2. Briefly, these sequences were first identified by MIRA (*56*). TCRs that reacted were further screened for enrichment in COVID-19–positive repertoires collected as part of immuneCODE (*15*) and compared with COVID-19–negative repertoires to remove TCRs that may be highly public or cross-reactive to common antigens. Individual response could be quantified by the number or frequency of SARS-CoV-2 TCRs seen after vaccination. TCRs were further analyzed to determine position within the spike protein based on the MIRA antigens.

Sequences of known variants were obtained from GISAID (www.gisaid.org) and aligned to known MIRA antigen locations. Antigens that contain any mutations observed in the B.1.1.7 or B.1.351 variants were labeled as potentially affected.

### Statistical analysis

For ICS analysis, significant differences between vaccination groups were determined by Mann-Whitney nonparametric two-tailed *t* tests with a 95% confidence interval. Significant differences between time points within each vaccine group were determined by Kruskal-Wallis nonparametric one-way analysis of variance (ANOVA) with Dunn’s test to correct for multiple comparisons. For all TCR analyses, significant differences were determined by one-way ANOVA followed by Sidak’s multiple comparisons tests. Raw data are available in data files S1 and S2.

## Notes

View/request a protocol for this paper from *Bio-protocol*.

## References

[1] J. M. Dan, J. Mateus, Y. Kato, K. M. Hastie, E. D. Yu, C. E. Faliti, A. Grifoni, S. I. Ramirez, S. Haupt, A. Frazier, C. Nakao, V. Rayaprolu, S. A. Rawlings, B. Peters, F. Krammer, V. Simon, E. O. Saphire, D. M. Smith, D. Weiskopf, A. Sette, S. Crotty, Immunological memory to SARS-CoV-2 assessed for up to 8 months after infection. Science 371, eabf4063 (2021). 33408181 10.1126/science.abf4063 PMC7919858

[2] F. Gallais, A. Velay, C. Nazon, M. J. Wendling, M. Partisani, J. Sibilia, S. Candon, S. Fafi-Kremer, Intrafamilial exposure to SARS-CoV-2 associated with cellular immune response without seroconversion, France. Emerg. Infect. Dis. 27, 113–121 (2021). 33261718 10.3201/eid2701.203611 PMC7774579

[3] C. Rydyznski Moderbacher, S. I. Ramirez, J. M. Dan, A. Grifoni, K. M. Hastie, D. Weiskopf, S. Belanger, R. K. Abbott, C. Kim, J. Choi, Y. Kato, E. G. Crotty, C. Kim, S. A. Rawlings, J. Mateus, L. P. V. Tse, A. Frazier, R. Baric, B. Peters, J. Greenbaum, E. Ollmann Saphire, D. M. Smith, A. Sette, S. Crotty, Antigen-specific adaptive immunity to SARS-CoV-2 in acute COVID-19 and associations with age and disease severity. Cell 183, 996–1012.e19 (2020). 33010815 10.1016/j.cell.2020.09.038 PMC7494270

[4] R. Zhou, K. K.-W. To, Y.-C. Wong, L. Liu, B. Zhou, X. Li, H. Huang, Y. Mo, T.-Y. Luk, T. T.-K. Lau, P. Yeung, W.-M. Chan, A. K.-L. Wu, K.-C. Lung, O. T.-Y. Tsang, W.-S. Leung, I. F.-N. Hung, K.-Y. Yuen, Z. Chen, Acute SARS-CoV-2 infection impairs dendritic cell and T cell responses. Immunity 53, 864–877.e5 (2020). 32791036 10.1016/j.immuni.2020.07.026 PMC7402670

[5] M. Liao, Y. Liu, J. Yuan, Y. Wen, G. Xu, J. Zhao, L. Cheng, J. Li, X. Wang, F. Wang, L. Liu, I. Amit, S. Zhang, Z. Zhang, Single-cell landscape of bronchoalveolar immune cells in patients with COVID-19. Nat. Med. 26, 842–844 (2020). 32398875 10.1038/s41591-020-0901-9

[6] U. Sahin, A. Muik, E. Derhovanessian, I. Vogler, L. M. Kranz, M. Vormehr, A. Baum, K. Pascal, J. Quandt, D. Maurus, S. Brachtendorf, V. Lorks, J. Sikorski, R. Hilker, D. Becker, A. K. Eller, J. Grutzner, C. Boesler, C. Rosenbaum, M. C. Kuhnle, U. Luxemburger, A. Kemmer-Bruck, D. Langer, M. Bexon, S. Bolte, K. Kariko, T. Palanche, B. Fischer, A. Schultz, P. Y. Shi, C. Fontes-Garfias, J. L. Perez, K. A. Swanson, J. Loschko, I. L. Scully, M. Cutler, W. Kalina, C. A. Kyratsous, D. Cooper, P. R. Dormitzer, K. U. Jansen, O. Tureci, COVID-19 vaccine BNT162b1 elicits human antibody and TH1 T cell responses. Nature 586, 594–599 (2020). 32998157 10.1038/s41586-020-2814-7

[7] E. J. Anderson, N. G. Rouphael, A. T. Widge, L. A. Jackson, P. C. Roberts, M. Makhene, J. D. Chappell, M. R. Denison, L. J. Stevens, A. J. Pruijssers, A. B. McDermott, B. Flach, B. C. Lin, N. A. Doria-Rose, S. O’Dell, S. D. Schmidt, K. S. Corbett, P. A. Swanson, M. Padilla, K. M. Neuzil, H. Bennett, B. Leav, M. Makowski, J. Albert, K. Cross, V. V. Edara, K. Floyd, M. S. Suthar, D. R. Martinez, R. Baric, W. Buchanan, C. J. Luke, V. K. Phadke, C. A. Rostad, J. E. Ledgerwood, B. S. Graham, J. H. Beigel, Safety and immunogenicity of SARS-CoV-2 mRNA-1273 vaccine in older adults. N. Engl. J. Med. 383, 2427–2438 (2020). 32991794 10.1056/NEJMoa2028436 PMC7556339

[8] J. Sadoff, M. Le Gars, G. Shukarev, D. Heerwegh, C. Truyers, A. M. de Groot, J. Stoop, S. Tete, W. Van Damme, I. Leroux-Roels, P. J. Berghmans, M. Kimmel, P. Van Damme, J. de Hoon, W. Smith, K. E. Stephenson, S. C. De Rosa, K. W. Cohen, M. J. McElrath, E. Cormier, G. Scheper, D. H. Barouch, J. Hendriks, F. Struyf, M. Douoguih, J. Van Hoof, H. Schuitemaker, Interim results of a phase 1-2a trial of Ad26.COV2.S Covid-19 vaccine. N. Engl. J. Med. 384, 1824–1835 (2021). 33440088 10.1056/NEJMoa2034201 PMC7821985

[9] C. Keech, G. Albert, I. Cho, A. Robertson, P. Reed, S. Neal, J. S. Plested, M. Zhu, S. Cloney-Clark, H. Zhou, G. Smith, N. Patel, M. B. Frieman, R. E. Haupt, J. Logue, M. McGrath, S. Weston, P. A. Piedra, C. Desai, K. Callahan, M. Lewis, P. Price-Abbott, N. Formica, V. Shinde, L. Fries, J. D. Lickliter, P. Griffin, B. Wilkinson, G. M. Glenn, Phase 1-2 trial of a SARS-CoV-2 recombinant spike protein nanoparticle vaccine. N. Engl. J. Med. 383, 2320–2332 (2020). 32877576 10.1056/NEJMoa2026920 PMC7494251

[10] L. A. Jackson, E. J. Anderson, N. G. Rouphael, P. C. Roberts, M. Makhene, R. N. Coler, M. P. McCullough, J. D. Chappell, M. R. Denison, L. J. Stevens, A. J. Pruijssers, A. McDermott, B. Flach, N. A. Doria-Rose, K. S. Corbett, K. M. Morabito, S. O’Dell, S. D. Schmidt, P. A. Swanson 2nd, M. Padilla, J. R. Mascola, K. M. Neuzil, H. Bennett, W. Sun, E. Peters, M. Makowski, J. Albert, K. Cross, W. Buchanan, R. Pikaart-Tautges, J. E. Ledgerwood, B. S. Graham, J. H. Beigel; mRNA-1273 Study Group, An mRNA vaccine against SARS-CoV-2 - preliminary report. N. Engl. J. Med. 383, 1920–1931 (2020). 32663912 10.1056/NEJMoa2022483 PMC7377258

[11] K. J. Ewer, J. R. Barrett, S. Belij-Rammerstorfer, H. Sharpe, R. Makinson, R. Morter, A. Flaxman, D. Wright, D. Bellamy, M. Bittaye, C. Dold, N. M. Provine, J. Aboagye, J. Fowler, S. E. Silk, J. Alderson, P. K. Aley, B. Angus, E. Berrie, S. Bibi, P. Cicconi, E. A. Clutterbuck, I. Chelysheva, P. M. Folegatti, M. Fuskova, C. M. Green, D. Jenkin, S. Kerridge, A. Lawrie, A. M. Minassian, M. Moore, Y. Mujadidi, E. Plested, I. Poulton, M. N. Ramasamy, H. Robinson, R. Song, M. D. Snape, R. Tarrant, M. Voysey, M. E. E. Watson, A. D. Douglas, A. V. S. Hill, S. C. Gilbert, A. J. Pollard, T. Lambe, A. Ali, E. Allen, M. Baker, E. Barnes, N. Borthwick, A. Boyd, C. Brown-O’Sullivan, J. Burgoyne, N. Byard, I. C. Puig, F. Cappuccini, J.-S. Cho, P. Cicconi, E. Clark, W. E. M. Crocker, M. S. Datoo, H. Davies, S. J. Dunachie, N. J. Edwards, S. C. Elias, J. Furze, C. Gilbride, S. A. Harris, S. H. C. Hodgson, M. M. Hou, S. Jackson, K. Jones, R. Kailath, L. King, C. W. Larkworthy, Y. Li, A. M. Lias, A. Linder, S. Lipworth, R. L. Ramon, M. Madhavan, E. Marlow, J. L. Marshall, A. J. Mentzer, H. Morrison, A. Noé, D. Pipini, D. Pulido-Gomez, F. R. Lopez, A. J. Ritchie, I. Rudiansyah, H. Sanders, A. Shea, S. Silk, A. J. Spencer, R. Tanner, Y. Themistocleous, M. Thomas, N. Tran, A. Truby, C. Turner, N. Turner, M. Ulaszewska, A. T. Worth, L. Kingham-Page, M. P. P. Alvarez, R. Anslow, L. Bates, K. Beadon, R. Beckley, A. Beveridge, E. M. Bijker, L. Blackwell, J. Burbage, S. Camara, M. Carr, R. Colin-Jones, R. Cooper, C. J. Cunningham, T. Demissie, C. D. Maso, N. Douglas, R. Drake-Brockman, R. E. Drury, K. R. W. Emary, S. Felle, S. Feng, K. J. Ford, E. Francis, L. Gracie, J. Hamlyn, B. Hanumunthadu, D. Harrison, T. C. Hart, S. Hawkins, J. Hill, E. Howe, N. Howell, E. Jones, J. Keen, S. Kelly, D. Kerr, L. Khan, J. Kinch, S. Koleva, E. A. Lees, A. Lelliott, X. Liu, S. Marinou, J. McEwan, E. Morey, G. Morshead, J. Muller, C. Munro, S. Murphy, P. Mweu, E. Nuthall, K. O’Brien, D. O’Connor, P. J. O’Reilly, B. Oguti, P. Osborne, N. Owino, K. Parker, K. Pfafferott, S. Provstgaard-Morys, H. Ratcliffe, T. Rawlinson, S. Rhead, H. Roberts, K. Sanders, L. Silva-Reyes, C. C. Smith, D. J. Smith, A. Szigeti, T. M. Thomas, A. Thompson, S. Tonks, R. Varughes, I. Vichos, L. Walker, C. White, R. White, X. L. Yao, C. P. Conlon, J. Frater, L. Cifuentes, I. Baleanu, E. Bolam, E. Boland, T. Brenner, B. E. Damratoski, C. Datta, O. E. Muhanna, R. Fisher, P. Galian-Rubio, G. Hodges, F. Jackson, S. Liu, L. Loew, R. Morgans, S. J. Morris, V. Olchawski, C. Oliveria, H. Parracho, E. R. Pabon, A. Tahiri-Alaoui, K. Taylor, P. Williams, D. Zizi, E. H. Arbe-Barnes, P. Baker, A. Batten, C. Downing, J. Drake, M. R. English, J. A. Henry, P. Iveson, A. Killen, T. B. King, J. P. J. Larwood, G. Mallett, K. Mansatta, N. Mirtorabi, M. Patrick-Smith, J. Perring, K. Radia, S. Roche, E. Schofield, R. T. W. Naude, J. Towner, N. Baker, K. R. Bewley, E. Brunt, K. R. Buttigieg, S. Charlton, N. S. Coombes, M. J. Elmore, K. Godwin, B. Hallis, D. Knott, L. McInroy, I. Shaik, K. Thomas, J. A. Tree, C. L. Blundell, M. Cao, D. Kelly, D. T. Skelly, A. Themistocleous, T. Dong, S. Field, E. Hamilton, E. Kelly, P. Klenerman, J. C. Knight, Y. Lie, C. Petropoulos, C. Sedik, T. Wrin, G. Meddaugh, Y. Peng, G. Screaton, E. Stafford, T cell and antibody responses induced by a single dose of ChAdOx1 nCoV-19 (AZD1222) vaccine in a phase 1/2 clinical trial. Nat. Med. 27, 270–278 (2021). 33335323 10.1038/s41591-020-01194-5

[12] P. M. Folegatti, K. J. Ewer, P. K. Aley, B. Angus, S. Becker, S. Belij-Rammerstorfer, D. Bellamy, S. Bibi, M. Bittaye, E. A. Clutterbuck, C. Dold, S. N. Faust, A. Finn, A. L. Flaxman, B. Hallis, P. Heath, D. Jenkin, R. Lazarus, R. Makinson, A. M. Minassian, K. M. Pollock, M. Ramasamy, H. Robinson, M. Snape, R. Tarrant, M. Voysey, C. Green, A. D. Douglas, A. V. S. Hill, T. Lambe, S. C. Gilbert, A. J. Pollard, J. Aboagye, K. Adams, A. Ali, E. Allen, J. L. Allison, R. Anslow, E. H. Arbe-Barnes, G. Babbage, K. Baillie, M. Baker, N. Baker, P. Baker, I. Baleanu, J. Ballaminut, E. Barnes, J. Barrett, L. Bates, A. Batten, K. Beadon, R. Beckley, E. Berrie, L. Berry, A. Beveridge, K. R. Bewley, E. M. Bijker, T. Bingham, L. Blackwell, C. L. Blundell, E. Bolam, E. Boland, N. Borthwick, T. Bower, A. Boyd, T. Brenner, P. D. Bright, C. Brown-O’Sullivan, E. Brunt, J. Burbage, S. Burge, K. R. Buttigieg, N. Byard, I. C. Puig, A. Calvert, S. Camara, M. Cao, F. Cappuccini, M. Carr, M. W. Carroll, V. Carter, K. Cathie, R. J. Challis, S. Charlton, I. Chelysheva, J.-S. Cho, P. Cicconi, L. Cifuentes, H. Clark, E. Clark, T. Cole, R. Colin-Jones, C. P. Conlon, A. Cook, N. S. Coombes, R. Cooper, C. A. Cosgrove, K. Coy, W. E. M. Crocker, C. J. Cunningham, B. E. Damratoski, L. Dando, M. S. Datoo, H. Davies, H. De Graaf, T. Demissie, C. Di Maso, I. Dietrich, T. Dong, F. R. Donnellan, N. Douglas, C. Downing, J. Drake, R. Drake-Brockman, R. E. Drury, S. J. Dunachie, N. J. Edwards, F. D. L. Edwards, C. J. Edwards, S. C. Elias, M. J. Elmore, K. R. W. Emary, M. R. English, S. Fagerbrink, S. Felle, S. Feng, S. Field, C. Fixmer, C. Fletcher, K. J. Ford, J. Fowler, P. Fox, E. Francis, J. Frater, J. Furze, M. Fuskova, E. Galiza, D. Gbesemete, C. Gilbride, K. Godwin, G. Gorini, L. Goulston, C. Grabau, L. Gracie, Z. Gray, L. B. Guthrie, M. Hackett, S. Halwe, E. Hamilton, J. Hamlyn, B. Hanumunthadu, I. Harding, S. A. Harris, A. Harris, D. Harrison, C. Harrison, T. C. Hart, L. Haskell, S. Hawkins, I. Head, J. A. Henry, J. Hill, S. H. C. Hodgson, M. M. Hou, E. Howe, N. Howell, C. Hutlin, S. Ikram, C. Isitt, P. Iveson, S. Jackson, F. Jackson, S. W. James, M. Jenkins, E. Jones, K. Jones, C. E. Jones, B. Jones, R. Kailath, K. Karampatsas, J. Keen, S. Kelly, D. Kelly, D. Kerr, S. Kerridge, L. Khan, U. Khan, A. Killen, J. Kinch, T. B. King, L. King, J. King, L. Kingham-Page, P. Klenerman, F. Knapper, J. C. Knight, D. Knott, S. Koleva, A. Kupke, C. W. Larkworthy, J. P. J. Larwood, A. Laskey, A. M. Lawrie, A. Lee, K. Y. N. Lee, E. A. Lees, H. Legge, A. Lelliott, N.-M. Lemm, A. M. Lias, A. Linder, S. Lipworth, X. Liu, S. Liu, R. L. Ramon, M. Lwin, F. Mabesa, M. Madhavan, G. Mallett, K. Mansatta, I. Marcal, S. Marinou, E. Marlow, J. L. Marshall, J. Martin, J. McEwan, L. McInroy, G. Meddaugh, A. J. Mentzer, N. Mirtorabi, M. Moore, E. Moran, E. Morey, V. Morgan, S. J. Morris, H. Morrison, G. Morshead, R. Morter, Y. F. Mujadidi, J. Muller, T. Munera-Huertas, C. Munro, A. Munro, S. Murphy, V. J. Munster, P. Mweu, A. Noé, F. L. Nugent, E. Nuthall, K. O’Brien, D. O’Connor, B. Oguti, J. L. Oliver, C. Oliveira, P. J. O’Reilly, M. Osborn, P. Osborne, C. Owen, D. Owens, N. Owino, M. Pacurar, K. Parker, H. Parracho, M. Patrick-Smith, V. Payne, J. Pearce, Y. Peng, M. P. P. Alvarez, J. Perring, K. Pfafferott, D. Pipini, E. Plested, H. Pluess-Hall, K. Pollock, I. Poulton, L. Presland, S. Provstgaard-Morys, D. Pulido, K. Radia, F. R. Lopez, J. Rand, H. Ratcliffe, T. Rawlinson, S. Rhead, A. Riddell, A. J. Ritchie, H. Roberts, J. Robson, S. Roche, C. Rohde, C. S. Rollier, R. Romani, I. Rudiansyah, S. Saich, S. Sajjad, S. Salvador, L. S. Riera, H. Sanders, K. Sanders, S. Sapaun, C. Sayce, E. Schofield, G. Screaton, B. Selby, C. Semple, H. R. Sharpe, I. Shaik, A. Shea, H. Shelton, S. Silk, L. Silva-Reyes, D. T. Skelly, H. Smee, C. C. Smith, D. J. Smith, R. Song, A. J. Spencer, E. Stafford, A. Steele, E. Stefanova, L. Stockdale, A. Szigeti, A. Tahiri-Alaoui, M. Tait, H. Talbot, R. Tanner, I. J. Taylor, V. Taylor, R. T. W. Naude, N. Thakur, Y. Themistocleous, A. Themistocleous, M. Thomas, T. M. Thomas, A. Thompson, S. Thomson-Hill, J. Tomlins, S. Tonks, J. Towner, N. Tran, J. A. Tree, A. Truby, K. Turkentine, C. Turner, N. Turner, S. Turner, T. Tuthill, M. Ulaszewska, R. Varughese, N. Van Doremalen, K. Veighey, M. K. Verheul, I. Vichos, E. Vitale, L. Walker, M. E. E. Watson, B. Welham, J. Wheat, C. White, R. White, A. T. Worth, D. Wright, S. Wright, X. L. Yao, Y. Yau, Safety and immunogenicity of the ChAdOx1 nCoV-19 vaccine against SARS-CoV-2: A preliminary report of a phase 1/2, single-blind, randomised controlled trial. Lancet 396, 467–478 (2020). 32702298 10.1016/S0140-6736(20)31604-4 PMC7445431

[13] M. N. Ramasamy, A. M. Minassian, K. J. Ewer, A. L. Flaxman, P. M. Folegatti, D. R. Owens, M. Voysey, P. K. Aley, B. Angus, G. Babbage, S. Belij-Rammerstorfer, L. Berry, S. Bibi, M. Bittaye, K. Cathie, H. Chappell, S. Charlton, P. Cicconi, E. A. Clutterbuck, R. Colin-Jones, C. Dold, K. R. W. Emary, S. Fedosyuk, M. Fuskova, D. Gbesemete, C. Green, B. Hallis, M. M. Hou, D. Jenkin, C. C. D. Joe, E. J. Kelly, S. Kerridge, A. M. Lawrie, A. Lelliott, M. N. Lwin, R. Makinson, N. G. Marchevsky, Y. Mujadidi, A. P. S. Munro, M. Pacurar, E. Plested, J. Rand, T. Rawlinson, S. Rhead, H. Robinson, A. J. Ritchie, A. L. Ross-Russell, S. Saich, N. Singh, C. C. Smith, M. D. Snape, R. Song, R. Tarrant, Y. Themistocleous, K. M. Thomas, T. L. Villafana, S. C. Warren, M. E. E. Watson, A. D. Douglas, A. V. S. Hill, T. Lambe, S. C. Gilbert, S. N. Faust, A. J. Pollard, J. Aboagye, K. Adams, A. Ali, E. R. Allen, L. Allen, J. L. Allison, F. Andritsou, R. Anslow, E. H. Arbe-Barnes, M. Baker, N. Baker, P. Baker, I. Baleanu, D. Barker, E. Barnes, J. R. Barrett, K. Barrett, L. Bates, A. Batten, K. Beadon, R. Beckley, D. Bellamy, A. Berg, L. Bermejo, E. Berrie, A. Beveridge, K. Bewley, E. M. Bijker, G. Birch, L. Blackwell, H. Bletchly, C. L. Blundell, S. R. Blundell, E. Bolam, E. Boland, D. Bormans, N. Borthwick, K. Boukas, T. Bower, F. Bowring, A. Boyd, T. Brenner, P. Brown, C. Brown-O’Sullivan, S. Bruce, E. Brunt, J. Burbage, J. Burgoyne, K. R. Buttigieg, N. Byard, I. C. Puig, S. Camara, M. Cao, F. Cappuccini, M. Carr, M. W. Carroll, P. Cashen, A. Cavey, J. Chadwick, R. Challis, D. Chapman, D. Charles, I. Chelysheva, J.-S. Cho, L. Cifuentes, E. Clark, S. Collins, C. P. Conlon, N. S. Coombes, R. Cooper, C. Cooper, W. E. M. Crocker, S. Crosbie, D. Cullen, C. Cunningham, F. Cuthbertson, B. E. Datoo, L. Dando, M. S. Datoo, C. Datta, H. Davies, S. Davies, E. J. Davis, J. Davis, D. Dearlove, T. Demissie, S. Di Marco, C. Di Maso, D. DiTirro, C. Docksey, T. Dong, F. R. Donnellan, N. Douglas, C. Downing, J. Drake, R. Drake-Brockman, R. E. Drury, S. J. Dunachie, C. J. Edwards, N. J. Edwards, O. El Muhanna, S. C. Elias, R. S. Elliott, M. J. Elmore, M. R. English, S. Felle, S. Feng, C. F. Da Silva, S. Field, R. Fisher, C. Fixmer, K. J. Ford, J. Fowler, E. Francis, J. Frater, J. Furze, P. Galian-Rubio, C. Galloway, H. Garlant, M. Gavrila, F. Gibbons, K. Gibbons, C. Gilbride, H. Gill, K. Godwin, K. Gordon-Quayle, G. Gorini, L. Goulston, C. Grabau, L. Gracie, N. Graham, N. Greenwood, O. Griffiths, G. Gupta, E. Hamilton, B. Hanumunthadu, S. A. Harris, T. Harris, D. Harrison, T. C. Hart, B. Hartnell, L. Haskell, S. Hawkins, J. A. Henry, M. H. Herrera, D. Hill, J. Hill, G. Hodges, S. H. C. Hodgson, K. L. Horton, E. Howe, N. Howell, J. Howes, B. Huang, J. Humphreys, H. E. Humphries, P. Iveson, F. Jackson, S. Jackson, S. Jauregui, H. Jeffers, B. Jones, C. E. Jones, E. Jones, K. Jones, A. Joshi, R. Kailath, J. Keen, D. M. Kelly, S. Kelly, D. Kelly, D. Kerr, L. Khan, B. Khozoee, A. Killen, J. Kinch, L. D. W. King, T. B. King, L. Kingham, P. Klenerman, J. C. Knight, D. Knott, S. Koleva, G. Lang, C. W. Larkworthy, J. P. J. Larwood, R. Law, A. Lee, K. Y. N. Lee, E. A. Lees, S. Leung, Y. Li, A. M. Lias, A. Linder, S. Lipworth, S. Liu, X. Liu, S. Lloyd, L. Loew, R. L. Ramon, M. Madhavan, D. O. Mainwaring, G. Mallett, K. Mansatta, S. Marinou, P. Marius, E. Marlow, P. Marriott, J. L. Marshall, J. Martin, S. Masters, J. McEwan, J. L. McGlashan, L. McInroy, N. McRobert, C. Megson, A. J. Mentzer, N. Mirtorabi, C. Mitton, M. Moore, M. Moran, E. Morey, R. Morgans, S. J. Morris, H. M. Morrison, G. Morshead, R. Morter, N. A. Moya, E. Mukhopadhyay, J. Muller, C. Munro, S. Murphy, P. Mweu, A. Noé, F. L. Nugent, K. O’Brien, D. O’Connor, B. Oguti, V. Olchawski, C. Oliveira, P. J. O’Reilly, P. Osborne, L. Owen, N. Owino, P. Papageorgiou, H. Parracho, K. Parsons, B. Patel, M. Patrick-Smith, Y. Peng, E. J. Penn, M. P. Peralta-Alvarez, J. Perring, C. Petropoulos, D. J. Phillips, D. Pipini, S. Pollard, I. Poulton, D. Pratt, L. Presland, P. C. Proud, S. Provstgaard-Morys, S. Pueschel, D. Pulido, R. Rabara, K. Radia, D. Rajapaska, F. R. Lopez, H. Ratcliffe, S. Rayhan, B. Rees, E. R. Pabon, H. Roberts, I. Robertson, S. Roche, C. S. Rollier, R. Romani, Z. Rose, I. Rudiansyah, S. Sabheha, S. Salvador, H. Sanders, K. Sanders, I. Satti, C. Sayce, A. B. Schmid, E. Schofield, G. Screaton, C. Sedik, S. Seddiqi, R. R. Segireddy, B. Selby, I. Shaik, H. R. Sharpe, R. Shaw, A. Shea, S. Silk, L. Silva-Reyes, D. T. Skelly, D. J. Smith, D. C. Smith, N. Smith, A. J. Spencer, L. Spoors, E. Stafford, I. Stamford, L. Stockdale, D. Stockley, L. V. Stockwell, M. Stokes, L. H. Strickland, A. Stuart, S. Sulaiman, E. Summerton, Z. Swash, A. Szigeti, A. Tahiri-Alaoui, R. Tanner, I. Taylor, K. Taylor, U. Taylor, R. te Water Naude, A. Themistocleous, M. Thomas, T. M. Thomas, A. Thompson, K. Thompson, V. Thornton-Jones, L. Tinh, A. Tomic, S. Tonks, J. Towner, N. Tran, J. A. Tree, A. Truby, C. Turner, R. Turner, M. Ulaszewska, R. Varughese, D. Verbart, M. K. Verheul, I. Vichos, L. Walker, M. E. Wand, B. Watkins, J. Welch, A. J. West, C. White, R. White, P. Williams, M. Woodyer, A. T. Worth, D. Wright, T. Wrin, X. L. Yao, D.-A. Zbarcea, D. Zizi, Safety and immunogenicity of ChAdOx1 nCoV-19 vaccine administered in a prime-boost regimen in young and old adults (COV002): A single-blind, randomised, controlled, phase 2/3 trial. Lancet 396, 1979–1993 (2020). 33220855 10.1016/S0140-6736(20)32466-1 PMC7674972

[14] M. Voysey, S. A. C. Clemens, S. A. Madhi, L. Y. Weckx, P. M. Folegatti, P. K. Aley, B. Angus, V. L. Baillie, S. L. Barnabas, Q. E. Bhorat, S. Bibi, C. Briner, P. Cicconi, A. M. Collins, R. Colin-Jones, C. L. Cutland, T. C. Darton, K. Dheda, C. J. A. Duncan, K. R. W. Emary, K. J. Ewer, L. Fairlie, S. N. Faust, S. Feng, D. M. Ferreira, A. Finn, A. L. Goodman, C. M. Green, C. A. Green, P. T. Heath, C. Hill, H. Hill, I. Hirsch, S. H. C. Hodgson, A. Izu, S. Jackson, D. Jenkin, C. C. D. Joe, S. Kerridge, A. Koen, G. Kwatra, R. Lazarus, A. M. Lawrie, A. Lelliott, V. Libri, P. J. Lillie, R. Mallory, A. V. A. Mendes, E. P. Milan, A. M. Minassian, A. McGregor, H. Morrison, Y. F. Mujadidi, A. Nana, P. J. O’Reilly, S. D. Padayachee, A. Pittella, E. Plested, K. M. Pollock, M. N. Ramasamy, S. Rhead, A. V. Schwarzbold, N. Singh, A. Smith, R. Song, M. D. Snape, E. Sprinz, R. K. Sutherland, R. Tarrant, E. C. Thomson, M. E. Török, M. Toshner, D. P. J. Turner, J. Vekemans, T. L. Villafana, M. E. E. Watson, C. J. Williams, A. D. Douglas, A. V. S. Hill, T. Lambe, S. C. Gilbert, A. J. Pollard, M. Aban, F. Abayomi, K. Abeyskera, J. Aboagye, M. Adam, K. Adams, J. Adamson, Y. A. Adelaja, G. Adewetan, S. Adlou, K. Ahmed, Y. Akhalwaya, S. Akhalwaya, A. Alcock, A. Ali, E. R. Allen, L. Allen, T. C. D. S. C. Almeida, M. P. S. Alves, F. Amorim, F. Andritsou, R. Anslow, M. Appleby, E. H. Arbe-Barnes, M. P. Ariaans, B. Arns, L. Arruda, P. Azi, L. Azi, G. Babbage, C. Bailey, K. F. Baker, M. Baker, N. Baker, P. Baker, L. Baldwin, I. Baleanu, D. Bandeira, A. Bara, M. A. S. Barbosa, D. Barker, G. D. Barlow, E. Barnes, A. S. Barr, J. R. Barrett, J. Barrett, L. Bates, A. Batten, K. Beadon, E. Beales, R. Beckley, S. Belij-Rammerstorfer, J. Bell, D. Bellamy, N. Bellei, S. Belton, A. Berg, L. Bermejo, E. Berrie, L. Berry, D. Berzenyi, A. Beveridge, K. R. Bewley, H. Bexhell, S. Bhikha, A. E. Bhorat, Z. E. Bhorat, E. Bijker, G. Birch, S. Birch, A. Bird, O. Bird, K. Bisnauthsing, M. Bittaye, K. Blackstone, L. Blackwell, H. Bletchly, C. L. Blundell, S. R. Blundell, P. Bodalia, B. C. Boettger, E. Bolam, E. Boland, D. Bormans, N. Borthwick, F. Bowring, A. Boyd, P. Bradley, T. Brenner, P. Brown, C. Brown, C. Brown-O’Sullivan, S. Bruce, E. Brunt, R. Buchan, W. Budd, Y. A. Bulbulia, M. Bull, J. Burbage, H. Burhan, A. Burn, K. R. Buttigieg, N. Byard, I. C. Puig, G. Calderon, A. Calvert, S. Camara, M. Cao, F. Cappuccini, J. R. Cardoso, M. Carr, M. W. Carroll, A. Carson-Stevens, Y. D. M. Carvalho, J. A. M. Carvalho, H. R. Casey, P. Cashen, T. Castro, L. C. Castro, K. Cathie, A. Cavey, J. Cerbino-Neto, J. Chadwick, D. Chapman, S. Charlton, I. Chelysheva, O. Chester, S. Chita, J.-S. Cho, L. Cifuentes, E. Clark, M. Clark, A. Clarke, E. A. Clutterbuck, S. L. K. Collins, C. P. Conlon, S. Connarty, N. Coombes, C. Cooper, R. Cooper, L. Cornelissen, T. Corrah, C. Cosgrove, T. Cox, W. E. M. Crocker, S. Crosbie, L. Cullen, D. Cullen, D. R. M. F. Cunha, C. Cunningham, F. C. Cuthbertson, S. N. F. Da Guarda, L. P. da Silva, B. E. Damratoski, Z. Danos, M. T. D. C. Dantas, P. Darroch, M. S. Datoo, C. Datta, M. Davids, S. L. Davies, H. Davies, E. Davis, J. Davis, J. Davis, M. M. D. De Nobrega, L. M. D. O. Kalid, D. Dearlove, T. Demissie, A. Desai, S. Di Marco, C. Di Maso, M. I. S. Dinelli, T. Dinesh, C. Docksey, C. Dold, T. Dong, F. R. Donnellan, T. D. Santos, T. G. dos Santos, E. P. D. Santos, N. Douglas, C. Downing, J. Drake, R. Drake-Brockman, K. Driver, R. Drury, S. J. Dunachie, B. S. Durham, L. Dutra, N. J. W. Easom, S. van Eck, M. Edwards, N. J. Edwards, O. M. El Muhanna, S. C. Elias, M. Elmore, M. English, A. Esmail, Y. M. Essack, E. Farmer, M. Farooq, M. Farrar, L. Farrugia, B. Faulkner, S. Fedosyuk, S. Felle, S. Feng, C. F. Da Silva, S. Field, R. Fisher, A. Flaxman, J. Fletcher, H. Fofie, H. Fok, K. J. Ford, J. Fowler, P. H. A. Fraiman, E. Francis, M. M. Franco, J. Frater, M. S. M. Freire, S. H. Fry, S. Fudge, J. Furze, M. Fuskova, P. Galian-Rubio, E. Galiza, H. Garlant, M. Gavrila, A. Geddes, K. A. Gibbons, C. Gilbride, H. Gill, S. Glynn, K. Godwin, K. Gokani, U. C. Goldoni, M. Goncalves, I. G. S. Gonzalez, J. Goodwin, A. Goondiwala, K. Gordon-Quayle, G. Gorini, J. Grab, L. Gracie, M. Greenland, N. Greenwood, J. Greffrath, M. M. Groenewald, L. Grossi, G. Gupta, M. Hackett, B. Hallis, M. Hamaluba, E. Hamilton, J. Hamlyn, D. Hammersley, A. T. Hanrath, B. Hanumunthadu, S. A. Harris, C. Harris, T. Harris, T. D. Harrison, D. Harrison, T. C. Hart, B. Hartnell, S. Hassan, J. Haughney, S. Hawkins, J. Hay, I. Head, J. Henry, M. H. Herrera, D. B. Hettle, J. Hill, G. Hodges, E. Horne, M. M. Hou, C. Houlihan, E. Howe, N. Howell, J. Humphreys, H. E. Humphries, K. Hurley, C. Huson, A. Hyder-Wright, C. Hyams, S. Ikram, A. Ishwarbhai, M. Ivan, P. Iveson, V. Iyer, F. Jackson, J. De Jager, S. Jaumdally, H. Jeffers, N. Jesudason, B. Jones, K. Jones, E. Jones, C. Jones, M. R. Jorge, A. Jose, A. Joshi, E. A. M. S. Júnior, J. Kadziola, R. Kailath, F. Kana, K. Karampatsas, M. Kasanyinga, J. Keen, E. J. Kelly, D. M. Kelly, D. Kelly, S. Kelly, D. Kerr, R. D. Á. Kfouri, L. Khan, B. Khozoee, S. Kidd, A. Killen, J. Kinch, P. Kinch, L. D. W. King, T. B. King, L. Kingham, P. Klenerman, F. Knapper, J. C. Knight, D. Knott, S. Koleva, M. Lang, G. Lang, C. W. Larkworthy, J. P. J. Larwood, R. Law, E. M. Lazarus, A. Leach, E. A. Lees, N.-M. Lemm, A. Lessa, S. Leung, Y. Li, A. M. Lias, K. Liatsikos, A. Linder, S. Lipworth, S. Liu, X. Liu, A. Lloyd, S. Lloyd, L. Loew, R. L. Ramon, L. Lora, V. Lowthorpe, K. Luz, J. C. MacDonald, G. MacGregor, M. Madhavan, D. O. Mainwaring, E. Makambwa, R. Makinson, M. Malahleha, R. Malamatsho, G. Mallett, K. Mansatta, T. Maoko, K. Mapetla, N. G. Marchevsky, S. Marinou, E. Marlow, G. N. Marques, P. Marriott, R. P. Marshall, J. L. Marshall, F. J. Martins, M. Masenya, M. Masilela, S. K. Masters, M. Mathew, H. Matlebjane, K. Matshidiso, O. Mazur, A. Mazzella, H. McCaughan, J. McEwan, J. McGlashan, L. McInroy, Z. McIntyre, D. McLenaghan, N. McRobert, S. McSwiggan, C. Megson, S. Mehdipour, W. Meijs, R. N. Á. Mendonça, A. J. Mentzer, N. Mirtorabi, C. Mitton, S. Mnyakeni, F. Moghaddas, K. Molapo, M. Moloi, M. Moore, M. I. Moraes-Pinto, M. Moran, E. Morey, R. Morgans, S. Morris, S. Morris, H. C. Morris, F. Morselli, G. Morshead, R. Morter, L. Mottal, A. Moultrie, N. Moya, M. Mpelembue, S. Msomi, Y. Mugodi, E. Mukhopadhyay, J. Muller, A. Munro, C. Munro, S. Murphy, P. Mweu, C. H. Myasaki, G. Naik, K. Naker, E. Nastouli, A. Nazir, B. Ndlovu, F. Neffa, C. Njenga, H. Noal, A. Noé, G. Novaes, F. L. Nugent, G. Nunes, K. O’Brien, D. O’Connor, M. Odam, S. Oelofse, B. Oguti, V. Olchawski, N. J. Oldfield, M. G. Oliveira, C. Oliveira, A. Oosthuizen, P. O’Reilly, P. Osborne, D. R. J. Owen, L. Owen, D. Owens, N. Owino, M. Pacurar, B. V. B. Paiva, E. M. F. Palhares, S. Palmer, S. Parkinson, H. M. R. T. Parracho, K. Parsons, D. Patel, B. Patel, F. Patel, K. Patel, M. Patrick-Smith, R. O. Payne, Y. Peng, E. J. Penn, A. Pennington, M. P. P. Alvarez, J. Perring, N. Perry, R. Perumal, S. Petkar, T. Philip, D. J. Phillips, J. Phillips, M. K. Phohu, L. Pickup, S. Pieterse, J. Piper, D. Pipini, M. Plank, J. Du Plessis, S. Pollard, J. Pooley, A. Pooran, I. Poulton, C. Powers, F. B. Presa, D. A. Price, V. Price, M. Primeira, P. C. Proud, S. Provstgaard-Morys, S. Pueschel, D. Pulido, S. Quaid, R. Rabara, A. Radford, K. Radia, D. Rajapaska, T. Rajeswaran, A. S. F. Ramos, F. R. Lopez, T. Rampling, J. Rand, H. Ratcliffe, T. Rawlinson, D. Rea, B. Rees, J. Reiné, M. Resuello-Dauti, E. R. Pabon, C. M. Ribiero, M. Ricamara, A. Richter, N. Ritchie, A. J. Ritchie, A. J. Robbins, H. Roberts, R. E. Robinson, H. Robinson, T. T. Rocchetti, B. P. Rocha, S. Roche, C. Rollier, L. Rose, A. L. R. Russell, L. Rossouw, S. Royal, I. Rudiansyah, S. Ruiz, S. Saich, C. Sala, J. Sale, A. M. Salman, N. Salvador, S. Salvador, M. Sampaio, A. D. Samson, A. Sanchez-Gonzalez, H. Sanders, K. Sanders, E. Santos, M. F. S. S. Guerra, I. Satti, J. E. Saunders, C. Saunders, A. Sayed, I. S. van der Loeff, A. B. Schmid, E. Schofield, G. Screaton, S. Seddiqi, R. R. Segireddy, R. Senger, S. Serrano, R. Shah, I. Shaik, H. E. Sharpe, K. Sharrocks, R. Shaw, A. Shea, A. Shepherd, J. G. Shepherd, F. Shiham, E. Sidhom, S. E. Silk, A. C. da Silva Moraes, G. Silva-Junior, L. Silva-Reyes, A. D. Silveira, M. B. V. Silveira, J. Sinha, D. T. Skelly, D. C. Smith, N. Smith, H. E. Smith, D. J. Smith, C. C. Smith, A. Soares, T. Soares, C. Solórzano, G. L. Sorio, K. Sorley, T. Sosa-Rodriguez, C. M. C. D. L. Souza, B. S. D. F. Souza, A. R. Souza, A. J. Spencer, F. Spina, L. Spoors, L. Stafford, I. Stamford, I. Starinskij, R. Stein, J. Steven, L. Stockdale, L. V. Stockwell, L. H. Strickland, A. C. Stuart, A. Sturdy, N. Sutton, A. Szigeti, A. Tahiri-Alaoui, R. Tanner, C. Taoushanis, A. W. Tarr, K. Taylor, U. Taylor, I. J. Taylor, J. Taylor, R. te Water Naude, Y. Themistocleous, A. Themistocleous, M. Thomas, K. Thomas, T. M. Thomas, A. Thombrayil, F. Thompson, A. Thompson, K. Thompson, A. Thompson, J. Thomson, V. Thornton-Jones, P. J. Tighe, L. A. Tinoco, G. Tiongson, B. Tladinyane, M. Tomasicchio, A. Tomic, S. Tonks, J. Towner, N. Tran, J. Tree, G. Trillana, C. Trinham, R. Trivett, A. Truby, B. L. Tsheko, A. Turabi, R. Turner, C. Turner, M. Ulaszewska, B. R. Underwood, R. Varughese, D. Verbart, M. Verheul, I. Vichos, T. Vieira, C. S. Waddington, L. Walker, E. Wallis, M. Wand, D. Warbick, T. Wardell, G. Warimwe, S. C. Warren, B. Watkins, E. Watson, S. Webb, A. Webb-Bridges, A. Webster, J. Welch, J. Wells, A. West, C. White, R. White, P. Williams, R. L. Williams, R. Winslow, M. Woodyer, A. T. Worth, D. Wright, M. Wroblewska, A. Yao, R. Zimmer, D. Zizi, P. Zuidewind, Safety and efficacy of the ChAdOx1 nCoV-19 vaccine (AZD1222) against SARS-CoV-2: An interim analysis of four randomised controlled trials in Brazil, South Africa, and the UK. Lancet 397, 99–111 (2021). 33306989 10.1016/S0140-6736(20)32661-1 PMC7723445

[15] T. M. Snyder, R. M. Gittelman, M. Klinger, D. H. May, E. J. Osborne, R. Taniguchi, H. J. Zahid, I. M. Kaplan, J. N. Dines, M. T. Noakes, R. Pandya, X. Chen, S. Elasady, E. Svejnoha, P. Ebert, M. W. Pesesky, P. De Almeida, H. O’Donnell, Q. DeGottardi, G. Keitany, J. Lu, A. Vong, R. Elyanow, P. Fields, J. Greissl, L. Baldo, S. Semprini, C. Cerchione, F. Nicolini, M. Mazza, O. M. Delmonte, K. Dobbs, R. Laguna-Goya, G. Carreno-Tarragona, S. Barrio, L. Imberti, A. Sottini, E. Quiros-Roldan, C. Rossi, A. Biondi, L. R. Bettini, M. D’Angio, P. Bonfanti, M. F. Tompkins, C. Alba, C. Dalgard, V. Sambri, G. Martinelli, J. D. Goldman, J. R. Heath, H. C. Su, L. D. Notarangelo, E. Paz-Artal, J. Martinez-Lopez, J. M. Carlson, H. S. Robins, Magnitude and dynamics of the T-cell response to SARS-CoV-2 infection at both individual and population levels. medRxiv 2020.07.31.20165647 [Preprint]. 17 September 2020. 10.1101/2020.07.31.20165647.

[16] P. Supasa, D. Zhou, W. Dejnirattisai, C. Liu, A. J. Mentzer, H. M. Ginn, Y. Zhao, H. M. E. Duyvesteyn, R. Nutalai, A. Tuekprakhon, B. Wang, G. C. Paesen, J. Slon-Campos, C. López-Camacho, B. Hallis, N. Coombes, K. R. Bewley, S. Charlton, T. S. Walter, E. Barnes, S. J. Dunachie, D. Skelly, S. F. Lumley, N. Baker, I. Shaik, H. E. Humphries, K. Godwin, N. Gent, A. Sienkiewicz, C. Dold, R. Levin, T. Dong, A. J. Pollard, J. C. Knight, P. Klenerman, D. Crook, T. Lambe, E. Clutterbuck, S. Bibi, A. Flaxman, M. Bittaye, S. Belij-Rammerstorfer, S. Gilbert, D. R. Hall, M. A. Williams, N. G. Paterson, W. James, M. W. Carroll, E. E. Fry, J. Mongkolsapaya, J. Ren, D. I. Stuart, G. R. Screaton, Reduced neutralization of SARS-CoV-2 B.1.1.7 variant by convalescent and vaccine sera. Cell 184, 2201–2211 (2021). 33743891 10.1016/j.cell.2021.02.033 PMC7891044

[17] A. T. Tan, M. Linster, C. W. Tan, N. Le Bert, W. N. Chia, K. Kunasegaran, Y. Zhuang, C. Y. L. Tham, A. Chia, G. J. D. Smith, B. Young, S. Kalimuddin, J. G. H. Low, D. Lye, L. F. Wang, A. Bertoletti, Early induction of functional SARS-CoV-2-specific T cells associates with rapid viral clearance and mild disease in COVID-19 patients. Cell Rep. 34, 108728 (2021). 33516277 10.1016/j.celrep.2021.108728 PMC7826084

[18] K. Haq, J. E. McElhaney, Immunosenescence: Influenza vaccination and the elderly. Curr. Opin. Immunol. 29, 38–42 (2014). 24769424 10.1016/j.coi.2014.03.008

[19] V. A. Fulginiti, J. J. Eller, A. W. Downie, C. H. Kempe, Altered reactivity to measles virus. Atypical measles in children previously immunized with inactivated measles virus vaccines. JAMA 202, 1075–1080 (1967). 6072745 10.1001/jama.202.12.1075

[20] H. W. Kim, J. G. Canchola, C. D. Brandt, G. Pyles, R. M. Chanock, K. Jensen, R. H. Parrott, Respiratory syncytial virus disease in infants despite prior administration of antigenic inactivated vaccine. Am. J. Epidemiol. 89, 422–434 (1969). 4305198 10.1093/oxfordjournals.aje.a120955

[21] B. S. Graham, G. S. Henderson, Y. W. Tang, X. Lu, K. M. Neuzil, D. G. Colley, Priming immunization determines T helper cytokine mRNA expression patterns in lungs of mice challenged with respiratory syncytial virus. J. Immunol. 151, 2032–2040 (1993). 8345194

[22] F. Yasui, C. Kai, M. Kitabatake, S. Inoue, M. Yoneda, S. Yokochi, R. Kase, S. Sekiguchi, K. Morita, T. Hishima, H. Suzuki, K. Karamatsu, Y. Yasutomi, H. Shida, M. Kidokoro, K. Mizuno, K. Matsushima, M. Kohara, Prior immunization with severe acute respiratory syndrome (SARS)-associated coronavirus (SARS-CoV) nucleocapsid protein causes severe pneumonia in mice infected with SARS-CoV. J. Immunol. 181, 6337–6348 (2008). 18941225 10.4049/jimmunol.181.9.6337

[23] A. S. Agrawal, X. Tao, A. Algaissi, T. Garron, K. Narayanan, B. H. Peng, R. B. Couch, C. T. Tseng, Immunization with inactivated Middle East respiratory syndrome coronavirus vaccine leads to lung immunopathology on challenge with live virus. Hum. Vaccin. Immunother. 12, 2351–2356 (2016). 27269431 10.1080/21645515.2016.1177688 PMC5027702

[24] E. Panagioti, P. Klenerman, L. N. Lee, S. H. van der Burg, R. Arens, Features of effective T cell-inducing vaccines against chronic viral infections. Front. Immunol. 9, 276 (2018). 29503649 10.3389/fimmu.2018.00276 PMC5820320

[25] P. A. Darrah, D. T. Patel, P. M. De Luca, R. W. Lindsay, D. F. Davey, B. J. Flynn, S. T. Hoff, P. Andersen, S. G. Reed, S. L. Morris, M. Roederer, R. A. Seder, Multifunctional TH1 cells define a correlate of vaccine-mediated protection against *Leishmania major*. Nat. Med. 13, 843–850 (2007). 17558415 10.1038/nm1592

[26] E. Barnes, A. Folgori, S. Capone, L. Swadling, S. Aston, A. Kurioka, J. Meyer, R. Huddart, K. Smith, R. Townsend, A. Brown, R. Antrobus, V. Ammendola, M. Naddeo, G. O’Hara, C. Willberg, A. Harrison, F. Grazioli, M. L. Esposito, L. Siani, C. Traboni, Y. Oo, D. Adams, A. Hill, S. Colloca, A. Nicosia, R. Cortese, P. Klenerman, Novel adenovirus-based vaccines induce broad and sustained T cell responses to HCV in man. Sci. Transl. Med. 4, 115ra111 (2012). 10.1126/scitranslmed.3003155 PMC3627207 22218690

[27] F. Bihl, C. Berger, J. V. Chisholm 3rd, L. M. Henry, B. Bertisch, A. Trojan, D. Nadal, R. F. Speck, M. Flepp, C. Brander, N. J. Mueller, H. I. V. C. S. Swiss, Cellular immune responses and disease control in acute AIDS-associated Kaposi’s sarcoma. AIDS 23, 1918–1922 (2009). 19609199 10.1097/QAD.0b013e3283300a91

[28] R. S. Akondy, N. D. Monson, J. D. Miller, S. Edupuganti, D. Teuwen, H. Wu, F. Quyyumi, S. Garg, J. D. Altman, C. Del Rio, H. L. Keyserling, A. Ploss, C. M. Rice, W. A. Orenstein, M. J. Mulligan, R. Ahmed, The yellow fever virus vaccine induces a broad and polyfunctional human memory CD8+ T cell response. J. Immunol. 183, 7919–7930 (2009). 19933869 10.4049/jimmunol.0803903 PMC3374958

[29] S. Kannanganat, B. G. Kapogiannis, C. Ibegbu, L. Chennareddi, P. Goepfert, H. L. Robinson, J. Lennox, R. R. Amara, Human immunodeficiency virus type 1 controllers but not noncontrollers maintain CD4 T cells coexpressing three cytokines. J. Virol. 81, 12071–12076 (2007). 17728221 10.1128/JVI.01261-07 PMC2168799

[30] E. Van Braeckel, I. Desombere, F. Clement, L. Vandekerckhove, C. Verhofstede, D. Vogelaers, G. Leroux-Roels, Polyfunctional CD4^+^ T cell responses in HIV-1-infected viral controllers compared with those in healthy recipients of an adjuvanted polyprotein HIV-1 vaccine. Vaccine 31, 3739–3746 (2013). 23707169 10.1016/j.vaccine.2013.05.021

[31] E. Vasileiou, C. R. Simpson, T. Shi, S. Kerr, U. Agrawal, A. Akbari, S. Bedston, J. Beggs, D. Bradley, A. Chuter, S. de Lusignan, A. B. Docherty, D. Ford, F. R. Hobbs, M. Joy, S. V. Katikireddi, J. Marple, C. McCowan, D. McGagh, J. McMenamin, E. Moore, J. L. Murray, J. Pan, L. Ritchie, S. A. Shah, S. Stock, F. Torabi, R. S. Tsang, R. Wood, M. Woolhouse, C. Robertson, A. Sheikh, Interim findings from first-dose mass COVID-19 vaccination roll-out and COVID-19 hospital admissions in Scotland: A national prospective cohort study. Lancet 397, 1646–1657 (2021). 33901420 10.1016/S0140-6736(21)00677-2 PMC8064669

[32] J. Lopez Bernal, N. Andrews, C. Gower, C. Robertson, J. Stowe, E. Tessier, R. Simmons, S. Cottrell, R. Roberts, M. O’Doherty, K. Brown, C. Cameron, D. Stockton, J. McMenamin, M. Ramsay, Effectiveness of the Pfizer-BioNTech and Oxford-AstraZeneca vaccines on covid-19 related symptoms, hospital admissions, and mortality in older adults in England: Test negative case-control study. BMJ 373, n1088 (2021). 33985964 10.1136/bmj.n1088 PMC8116636

[33] Public Health England, COVID-19 vaccine surveillance report week 20; https://assets.publishing.service.gov.uk/government/uploads/system/uploads/attachment_data/file/988193/Vaccine_surveillance_report_-_week_20.pdf [accessed May 2021].

[34] A. Addetia, K. H. D. Crawford, A. Dingens, H. Zhu, P. Roychoudhury, M. L. Huang, K. R. Jerome, J. D. Bloom, A. L. Greninger, Neutralizing antibodies correlate with protection from SARS-CoV-2 in humans during a fishery vessel outbreak with a high attack rate. J. Clin. Microbiol. 58, e02107–e02120 (2020). 32826322 10.1128/JCM.02107-20 PMC7587101

[35] S. Crotty, A brief history of T cell help to B cells. Nat. Rev. Immunol. 15, 185–189 (2015). 25677493 10.1038/nri3803 PMC4414089

[36] A. Grifoni, D. Weiskopf, S. I. Ramirez, J. Mateus, J. M. Dan, C. R. Moderbacher, S. A. Rawlings, A. Sutherland, L. Premkumar, R. S. Jadi, D. Marrama, A. M. de Silva, A. Frazier, A. F. Carlin, J. A. Greenbaum, B. Peters, F. Krammer, D. M. Smith, S. Crotty, A. Sette, Targets of T cell responses to SARS-CoV-2 coronavirus in humans with COVID-19 disease and unexposed individuals. Cell 181, 1489–1501.e15 (2020). 32473127 10.1016/j.cell.2020.05.015 PMC7237901

[37] S. Crotty, T follicular helper cell biology: A decade of discovery and diseases. Immunity 50, 1132–1148 (2019). 31117010 10.1016/j.immuni.2019.04.011 PMC6532429

[38] J. A. Juno, H. X. Tan, W. S. Lee, A. Reynaldi, H. G. Kelly, K. Wragg, R. Esterbauer, H. E. Kent, C. J. Batten, F. L. Mordant, N. A. Gherardin, P. Pymm, M. H. Dietrich, N. E. Scott, W. H. Tham, D. I. Godfrey, K. Subbarao, M. P. Davenport, S. J. Kent, A. K. Wheatley, Humoral and circulating follicular helper T cell responses in recovered patients with COVID-19. Nat. Med. 26, 1428–1434 (2020). 32661393 10.1038/s41591-020-0995-0

[39] S. Santra, H. X. Liao, R. Zhang, M. Muldoon, S. Watson, W. Fischer, J. Theiler, J. Szinger, H. Balachandran, A. Buzby, D. Quinn, R. J. Parks, C. Y. Tsao, A. Carville, K. G. Mansfield, G. N. Pavlakis, B. K. Felber, B. F. Haynes, B. T. Korber, N. L. Letvin, Mosaic vaccines elicit CD8+ T lymphocyte responses that confer enhanced immune coverage of diverse HIV strains in monkeys. Nat. Med. 16, 324–328 (2010). 20173754 10.1038/nm.2108 PMC2834806

[40] D. H. Barouch, K. L. O’Brien, N. L. Simmons, S. L. King, P. Abbink, L. F. Maxfield, Y. H. Sun, A. La Porte, A. M. Riggs, D. M. Lynch, S. L. Clark, K. Backus, J. R. Perry, M. S. Seaman, A. Carville, K. G. Mansfield, J. J. Szinger, W. Fischer, M. Muldoon, B. Korber, Mosaic HIV-1 vaccines expand the breadth and depth of cellular immune responses in rhesus monkeys. Nat. Med. 16, 319–323 (2010). 20173752 10.1038/nm.2089 PMC2834868

[41] M. A. Martins, L. Gonzalez-Nieto, M. J. Ricciardi, V. K. Bailey, C. M. Dang, G. F. Bischof, N. Pedreno-Lopez, M. G. Pauthner, D. R. Burton, C. L. Parks, P. Earl, B. Moss, E. G. Rakasz, J. D. Lifson, R. C. Desrosiers, D. I. Watkins, Rectal acquisition of simian immunodeficiency virus (SIV) SIVmac239 infection despite vaccine-induced immune responses against the entire SIV proteome. J. Virol. 94, e00979-20 (2020). 33028714 10.1128/JVI.00979-20 PMC7925177

[42] A. Nelde, T. Bilich, J. S. Heitmann, Y. Maringer, H. R. Salih, M. Roerden, M. Lubke, J. Bauer, J. Rieth, M. Wacker, A. Peter, S. Horber, B. Traenkle, P. D. Kaiser, U. Rothbauer, M. Becker, D. Junker, G. Krause, M. Strengert, N. Schneiderhan-Marra, M. F. Templin, T. O. Joos, D. J. Kowalewski, V. Stos-Zweifel, M. Fehr, A. Rabsteyn, V. Mirakaj, J. Karbach, E. Jager, M. Graf, L. C. Gruber, D. Rachfalski, B. Preuss, I. Hagelstein, M. Marklin, T. Bakchoul, C. Gouttefangeas, O. Kohlbacher, R. Klein, S. Stevanovic, H. G. Rammensee, J. S. Walz, SARS-CoV-2-derived peptides define heterologous and COVID-19-induced T cell recognition. Nat. Immunol. 22, 74–85 (2021). 32999467 10.1038/s41590-020-00808-x

[43] H. Tegally, E. Wilkinson, M. Giovanetti, A. Iranzadeh, V. Fonseca, J. Giandhari, D. Doolabh, S. Pillay, E. J. San, N. Msomi, K. Mlisana, A. von Gottberg, S. Walaza, M. Allam, A. Ismail, T. Mohale, A. J. Glass, S. Engelbrecht, G. Van Zyl, W. Preiser, F. Petruccione, A. Sigal, D. Hardie, G. Marais, N. Y. Hsiao, S. Korsman, M. A. Davies, L. Tyers, I. Mudau, D. York, C. Maslo, D. Goedhals, S. Abrahams, O. Laguda-Akingba, A. Alisoltani-Dehkordi, A. Godzik, C. K. Wibmer, B. T. Sewell, J. Lourenco, L. C. J. Alcantara, S. L. Kosakovsky Pond, S. Weaver, D. Martin, R. J. Lessells, J. N. Bhiman, C. Williamson, T. de Oliveira, Detection of a SARS-CoV-2 variant of concern in South Africa. Nature 592, 438–443 (2021). 33690265 10.1038/s41586-021-03402-9

[44] B. Meng, S. A. Kemp, G. Papa, R. Datir, I. Ferreira, S. Marelli, W. T. Harvey, S. Lytras, A. Mohamed, G. Gallo, N. Thakur, D. A. Collier, P. Mlcochova, C.-G. U. Consortium, L. M. Duncan, A. M. Carabelli, J. C. Kenyon, A. M. Lever, A. De Marco, C. Saliba, K. Culap, E. Cameroni, N. J. Matheson, L. Piccoli, D. Corti, L. C. James, D. L. Robertson, D. Bailey, R. K. Gupta, Recurrent emergence of SARS-CoV-2 spike deletion H69/V70 and its role in the alpha variant B.1.1.7. Cell Rep. 35, 109292 (2021). 34166617 10.1016/j.celrep.2021.109292 PMC8185188

[45] R. J. Fischer, N. van Doremalen, D. R. Adney, C. K. Yinda, J. R. Port, M. G. Holbrook, J. E. Schulz, B. N. Williamson, T. Thomas, K. Barbian, S. L. Anzick, S. Ricklefs, B. J. Smith, D. Long, C. Martens, G. Saturday, E. de Wit, S. C. Gilbert, T. Lambe, V. J. Munster, ChAdOx1 nCoV-19 (AZD1222) protects against SARS-CoV-2 B.1.351 and B.1.1.7. bioRxiv 2021.03.11.435000 [Preprint]. 11 March 2021. 10.1101/2021.03.11.435000.

[46] K. R. W. Emary, T. Golubchik, P. K. Aley, C. V. Ariani, B. Angus, S. Bibi, B. Blane, D. Bonsall, P. Cicconi, S. Charlton, E. A. Clutterbuck, A. M. Collins, T. Cox, T. C. Darton, C. Dold, A. D. Douglas, C. J. A. Duncan, K. J. Ewer, A. L. Flaxman, S. N. Faust, D. M. Ferreira, S. Feng, A. Finn, P. M. Folegatti, M. Fuskova, E. Galiza, A. L. Goodman, C. M. Green, C. A. Green, M. Greenland, B. Hallis, P. T. Heath, J. Hay, H. C. Hill, D. Jenkin, S. Kerridge, R. Lazarus, V. Libri, P. J. Lillie, C. Ludden, N. G. Marchevsky, A. M. Minassian, A. C. McGregor, Y. F. Mujadidi, D. J. Phillips, E. Plested, K. M. Pollock, H. Robinson, A. Smith, R. Song, M. D. Snape, R. K. Sutherland, E. C. Thomson, M. Toshner, D. P. J. Turner, J. Vekemans, T. L. Villafana, C. J. Williams, A. V. S. Hill, T. Lambe, S. C. Gilbert, M. Voysey, M. N. Ramasamy, A. J. Pollard; COVID-19 Genomics UK Consortium; AMPHEUS Project; Oxford COVID-19 Vaccine Trial Group, Efficacy of ChAdOx1 nCoV-19 (AZD1222) vaccine against SARS-CoV-2 variant of concern 202012/01 (B.1.1.7): An exploratory analysis of a randomised controlled trial. Lancet 397, 1351–1362 (2021). 33798499 10.1016/S0140-6736(21)00628-0 PMC8009612

[47] A. Tarke, J. Sidney, N. Methot, E. D. Yu, Y. Zhang, J. M. Dan, B. Goodwin, P. Rubiro, A. Sutherland, E. Wang, A. Frazier, S. I. Ramirez, S. A. Rawlings, D. M. Smith, R. da Silva Antunes, B. Peters, R. H. Scheuermann, D. Weiskopf, S. Crotty, A. Grifoni, A. Sette, Impact of SARS-CoV-2 variants on the total CD4^+^ and CD8^+^ T cell reactivity in infected or vaccinated individuals. Cell. Rep. Med. 2, 100355 (2021). 34230917 10.1016/j.xcrm.2021.100355 PMC8249675

[48] A. D. Redd, A. Nardin, H. Kared, E. M. Bloch, A. Pekosz, O. Laeyendecker, B. Abel, M. Fehlings, T. C. Quinn, A. A. R. Tobian, CD8+ T-cell responses in COVID-19 convalescent individuals target conserved epitopes from multiple prominent SARS-CoV-2 circulating variants. Open Forum Infect. Dis. 8, ofab143 (2021). 34322559 10.1093/ofid/ofab143 PMC8083629

[49] G. Alter, J. Yu, J. Liu, A. Chandrashekar, E. N. Borducchi, L. H. Tostanoski, K. McMahan, C. Jacob-Dolan, D. R. Martinez, A. Chang, T. Anioke, M. Lifton, J. Nkolola, K. E. Stephenson, C. Atyeo, S. Shin, P. Fields, I. Kaplan, H. Robins, F. Amanat, F. Krammer, R. S. Baric, M. Le Gars, J. Sadoff, A. M. de Groot, D. Heerwegh, F. Struyf, M. Douoguih, J. van Hoof, H. Schuitemaker, D. H. Barouch, Immunogenicity of Ad26.COV2.S vaccine against SARS-CoV-2 variants in humans. Nature 596, 268–272 (2021). 34107529 10.1038/s41586-021-03681-2 PMC8357629

[50] A. Sette, S. Crotty, Adaptive immunity to SARS-CoV-2 and COVID-19. Cell 184, 861–880 (2021). 33497610 10.1016/j.cell.2021.01.007 PMC7803150

[51] P. A. Swanson 2nd, R. A. Seder, OMIP-067: 28-Color flow cytometry panel to evaluate human T-cell phenotype and function. Cytometry A 97, 1032–1036 (2020). 32677151 10.1002/cyto.a.24189

[52] G. Monaco, H. Chen, M. Poidinger, J. Chen, J. P. de Magalhaes, A. Larbi, flowAI: Automatic and interactive anomaly discerning tools for flow cytometry data. Bioinformatics 32, 2473–2480 (2016). 27153628 10.1093/bioinformatics/btw191

[53] H. Robins, C. Desmarais, J. Matthis, R. Livingston, J. Andriesen, H. Reijonen, C. Carlson, G. Nepom, C. Yee, K. Cerosaletti, Ultra-sensitive detection of rare T cell clones. J. Immunol. Methods 375, 14–19 (2012). 21945395 10.1016/j.jim.2011.09.001 PMC3721519

[54] H. S. Robins, P. V. Campregher, S. K. Srivastava, A. Wacher, C. J. Turtle, O. Kahsai, S. R. Riddell, E. H. Warren, C. S. Carlson, Comprehensive assessment of T-cell receptor β-chain diversity in αβ T cells. Blood 114, 4099–4107 (2009). 19706884 10.1182/blood-2009-04-217604 PMC2774550

[55] C. S. Carlson, R. O. Emerson, A. M. Sherwood, C. Desmarais, M.-W. Chung, J. M. Parsons, M. S. Steen, M. A. LaMadrid-Herrmannsfeldt, D. W. Williamson, R. J. Livingston, D. Wu, B. L. Wood, M. J. Rieder, H. Robins, Using synthetic templates to design an unbiased multiplex PCR assay. Nat. Commun. 4, 2680 (2013). 24157944 10.1038/ncomms3680

[56] M. Klinger, F. Pepin, J. Wilkins, T. Asbury, T. Wittkop, J. Zheng, M. Moorhead, M. Faham, Multiplex identification of antigen-specific T cell receptors using a combination of immune assays and immune receptor sequencing. PLOS ONE 10, e0141561 (2015). 26509579 10.1371/journal.pone.0141561 PMC4624875

