# Supplementary material for: AZD1222/ChAdOx1 nCoV-19 vaccination induces a polyfunctional spike protein–specific TH1 response with a diverse TCR repertoire
Source: Sci Transl Med. 2021 Nov 17;13(620):eabj7211. doi: 10.1126/scitranslmed.abj7211 (PMC9924073; doi:10.1126/scitranslmed.abj7211)
Supplement: Supplementary file 1 — Figs. S1 to S9 Tables S1 to S6 [file scitranslmed.abj7211_sm.pdf]

Science Translational Medicine
AAAS

Supplementary Materials for

AZD1222/ChAdOx1 nCoV-19 vaccination induces a polyfunctional spike protein–specific T_H1 response with a diverse TCR repertoire

Phillip A. Swanson II *et al.*

Corresponding author: Phillip A. Swanson, phil.swanson@nih.gov; Elizabeth J. Kelly, beth.kelly@astrazeneca.com

Sci. Transl. Med. **13**, eabj7211 (2021)
DOI: 10.1126/scitranslmed.abj7211

The PDF file includes:

Figs. S1 to S9
Tables S1 to S6

Other Supplementary Material for this manuscript includes the following:

Data files S1 and S2

Supplementary Materials

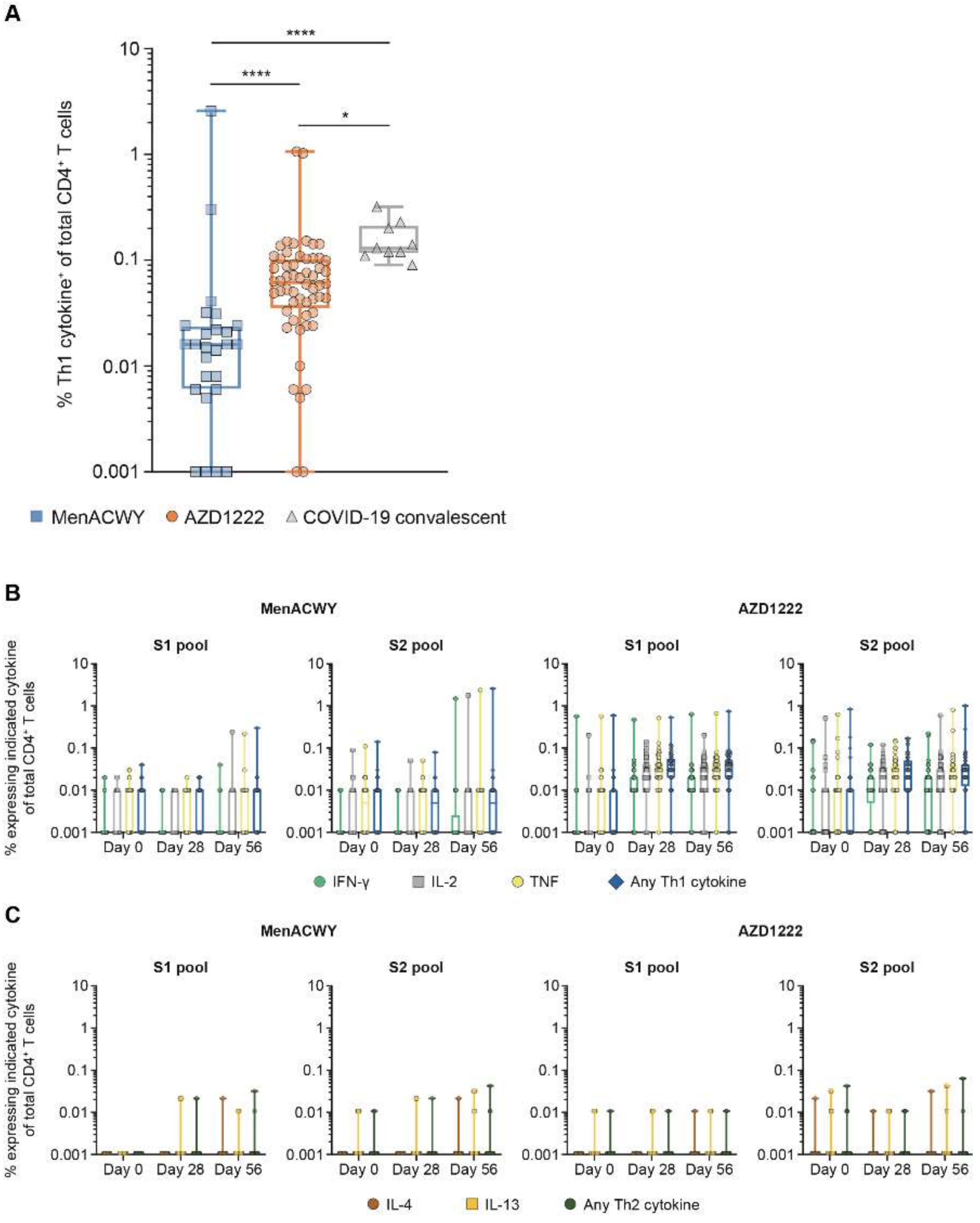

Fig. S1. Spike protein-specific CD4$^+$ T cell responses following vaccination with AZD1222 or MenACWY. (**A**) Peripheral blood mononuclear cells (PBMCs) from human participants vaccinated with AZD1222 or meningococcal conjugate vaccine, MenACWY, at Day 56 post vaccination and PBMCs from coronavirus disease 2019 **(**COVID-19) convalescent humans were stimulated with severe acute respiratory syndrome coronavirus 2 (SARS-CoV-2) spike protein peptide pools, and the intracellular cytokine response was measured. Frequencies of total spike protein-specific CD4$^+$ T cells producing any combination of the T cell helper type 1 (Th1) cytokines, interferon (IFN)-γ, interleukin (IL)-2, or tumor necrosis factor (TNF), following vaccination with AZD1222 or MenACWY, or from COVID-19 convalescent patients. Responses to each peptide pool were combined to determine the total spike protein-specific response. In the box and whisker plots, the horizontal line represents median, boxes represent interquartile range, whiskers extend to the min and max, and symbols represent each participant. Significant differences between each group were determined by Kruskal-Wallis test with Dunn's test to correct for multiple comparisons. All comparisons were not significant unless stated as significant; *$p<0.05$, ****$p<0.0001$. (**B and C**) To determine spike protein-specific responses, PBMCs were stimulated with two different peptide pools covering the entire SARS-CoV-2 spike protein (S1 and S2 pools). The responses for both pools were combined for Fig. 1 to determine the overall spike protein-specific response. Shown here are the data from Fig. 1, deconstructed into the T cell responses to each individual peptide pool. Th1 (B), and Th2 (C) CD4$^+$ T cell cytokine responses are shown following stimulation with the indicated peptide pool from AZD1222 (left) or MenACWY (right) vaccinated participants. In the box and whisker plots, the horizontal line represents median, boxes represent interquartile range, whiskers extend to the min and max, and symbols represent each participant.

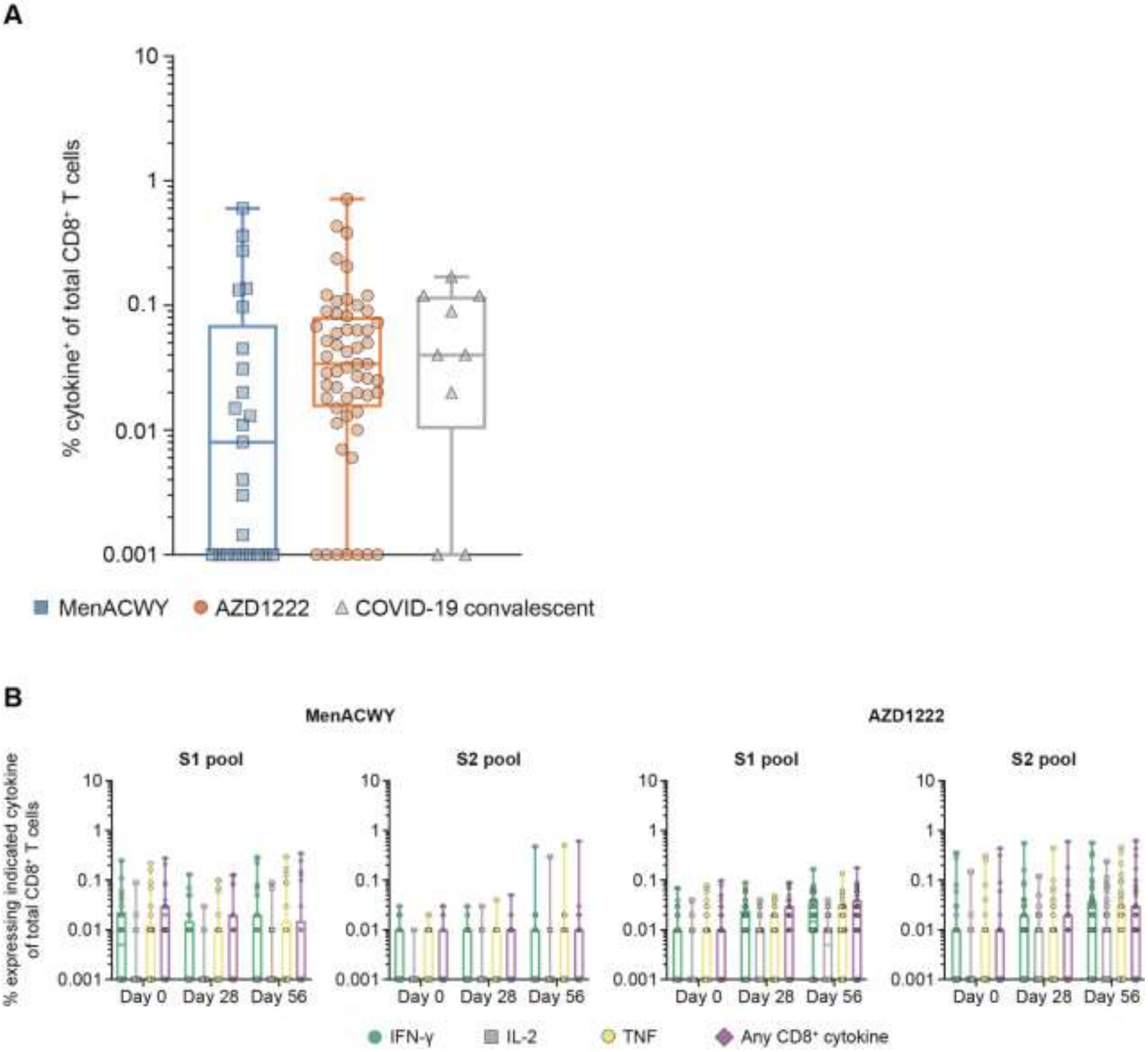
A
10
1
0.1
0.01
0.001
% cytokine+ of total CD8+ T cells
MenACWY
AZD1222
COVID-19 convalescent
B
MenACWY
AZD1222
S1 pool
S2 pool
S1 pool
S2 pool
% expressing indicated cytokine of total CD8+ T cells
Day 0
Day 28
Day 56
IFN-γ
IL-2
TNF
Any CD8+ cytokine

Fig. S2. Spike protein-specific CD8$^+$ T cell responses following vaccination with AZD1222 or MenACWY. (**A**) PBMCs from human participants vaccinated with AZD1222 or MenACWY at Day 56 post vaccination and PBMCs from COVID-19 convalescent humans were stimulated with SARS-CoV-2 spike protein peptide pools, and the intracellular cytokine response was measured. Frequencies of total spike protein-specific CD8$^+$ T cells producing any combination of IFN-γ, IL-2, or TNF, following vaccination with AZD1222 or MenACWY, or from COVID-19 convalescent patients. Responses to each peptide pool were combined to determine the total spike protein-specific response. In the box and whisker plots, the horizontal line represents median, boxes represent interquartile range, whiskers extend to the min and max, and symbols represent each participant. Significant differences between each group were determined by Kruskal-Wallis test with Dunn's test to correct for multiple comparisons. All comparisons were not significant unless stated as significant; *$p<0.05$, ****$p<0.0001$. (**B**) To determine spike protein-specific responses, PBMCs were stimulated with two different peptide pools covering the entire SARS-CoV-2 spike protein (S1 and S2 pools). The responses for both pools were combined for Fig. 1 to determine the overall spike protein-specific response. Shown here are the data from Fig. 1, deconstructed into the T cell responses to each individual peptide pool. CD8$^+$ T cell cytokine responses are shown following stimulation with the indicated peptide pool from AZD1222 (left) or MenACWY (right) vaccinated participants. In the box and whisker plots, the horizontal line represents median, boxes represent interquartile range, whiskers extend to the min and max, and symbols represent each participant.

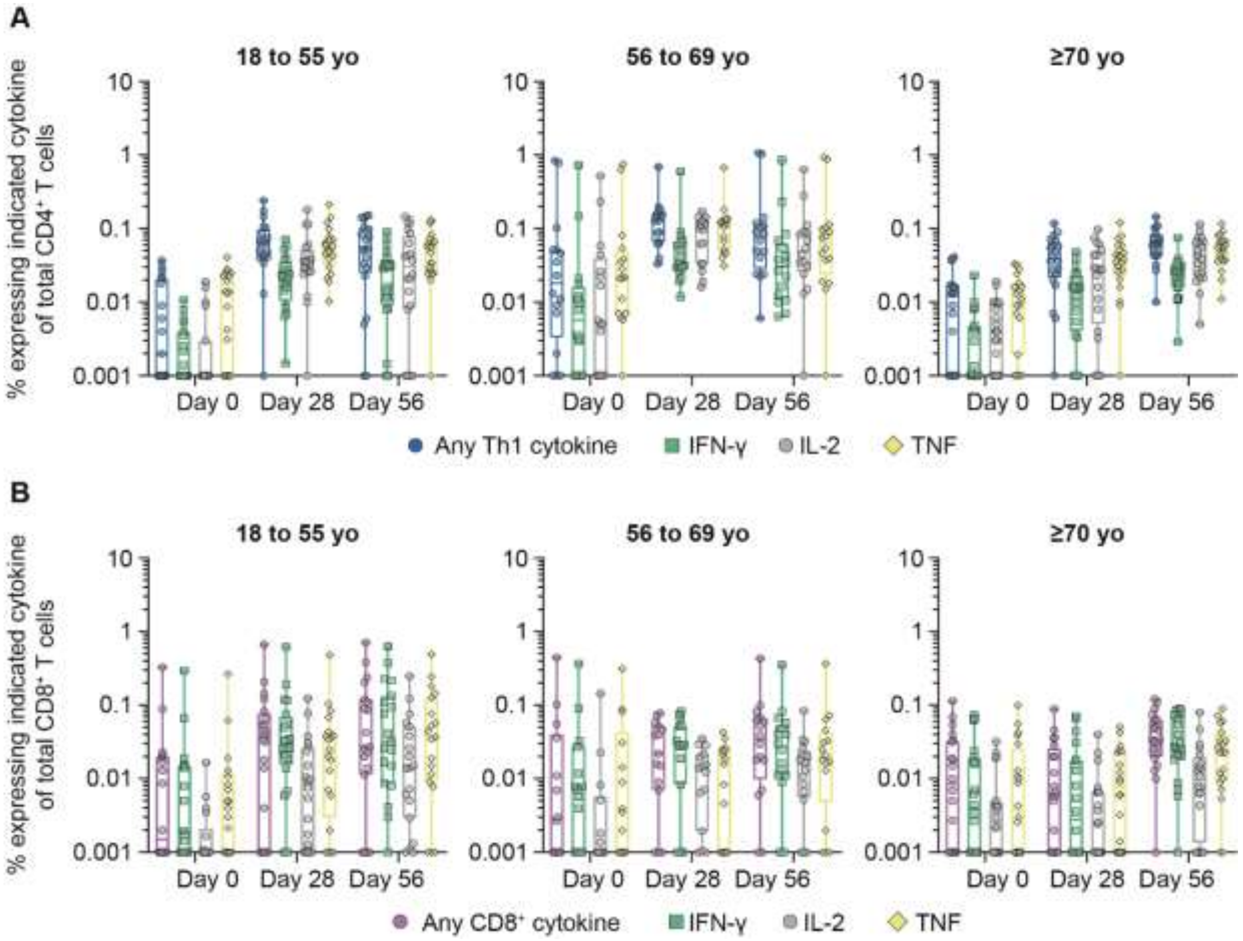

Fig. S3. Individual age-specific T cell responses to AZD1222 vaccination. (**A and B**) Frequencies of $CD4^+$ T cells (A) or $CD8^+$ T cells (B) from participants within each age cohort producing IFN-γ, IL-2, TNF, or any combination of these cytokines are shown for the indicated timepoints following stimulation with SARS-CoV-2 spike peptide pools. In the box and whisker plots, the horizontal line represents median, boxes represent interquartile range, whiskers extend to the min and max, and symbols represent each participant. yo, years old.

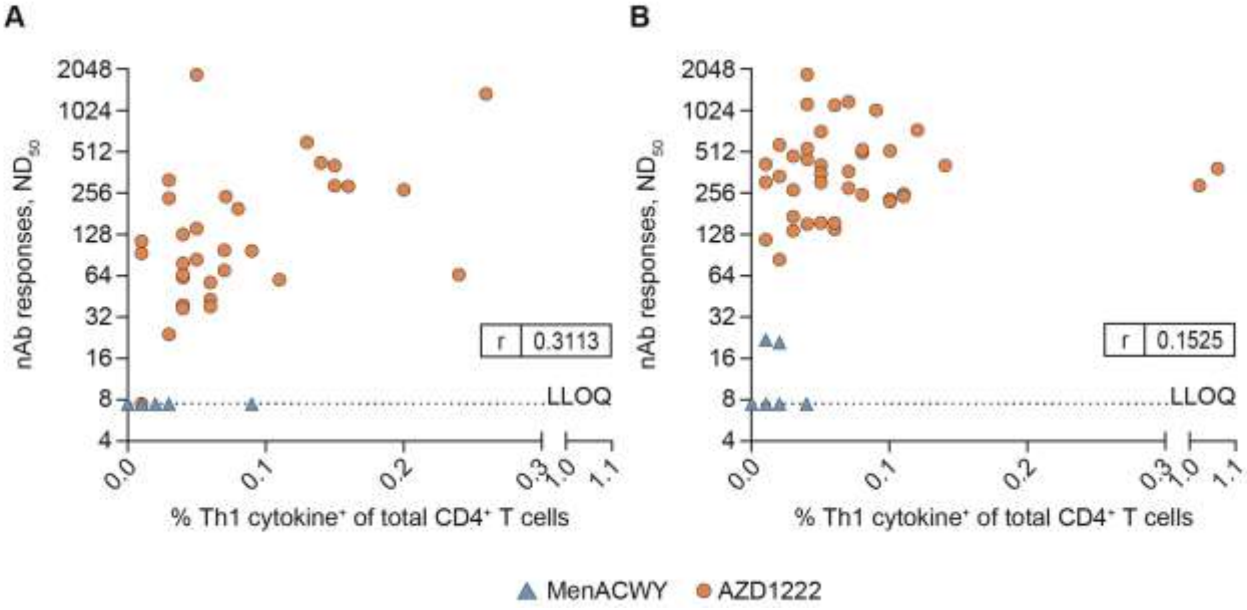

Fig. S4. Correlation between neutralizing antibody responses and CD4+ T cell responses. (**A and B**) SARS-CoV-2 neutralizing antibody titers (nAb, as determined by a live virus microneutralization assay) from serum of MenACWY or AZD1222 vaccinated study participants at 28 days post vaccination (A) or 56 days post vaccination (B) compared to frequencies of CD4+ T cells expressing any Th1 cytokine response. ND_{50}, 50% neutralizing dose; LLOQ, lower limit of quantification.

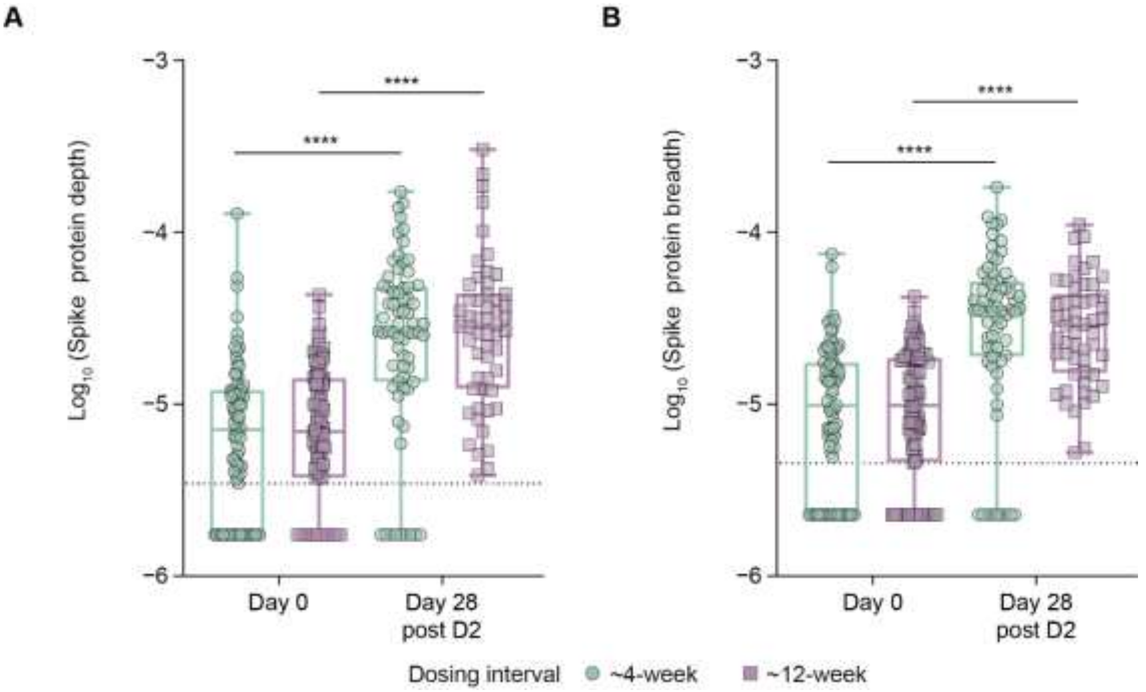

Fig. S5. Spike protein-specific T cell receptor (TCR) depth and breadth following vaccination with AZD1222 at 4-week and 12-week second dose schedules.
(**A and B**) Spike protein-specific TCR depth (A) and breadth (B) following vaccination with AZD1222 at approximately 4-week (18–60 days) and approximately 12-week (61–130 days) second dose (D2) schedules. Data are $\log_{10}$ transformed, where all zero values have been converted to half of the lowest non-zero value. Depth indicates SARS-CoV-2 associated T cells of total T cells. Breadth indicates SARS-CoV-2 associated unique TCRs of total unique TCRs. In the box and whisker plots, the horizontal line represents median, boxes represent interquartile range, whiskers extend to the min and max, and symbols represent each participant. The dotted lines represent the lower detected value. Significant differences determined by one-way ANOVA followed by Sidak's multiple comparisons tests. All comparisons are not significant unless stated as significant; ****$p<0.0001$.

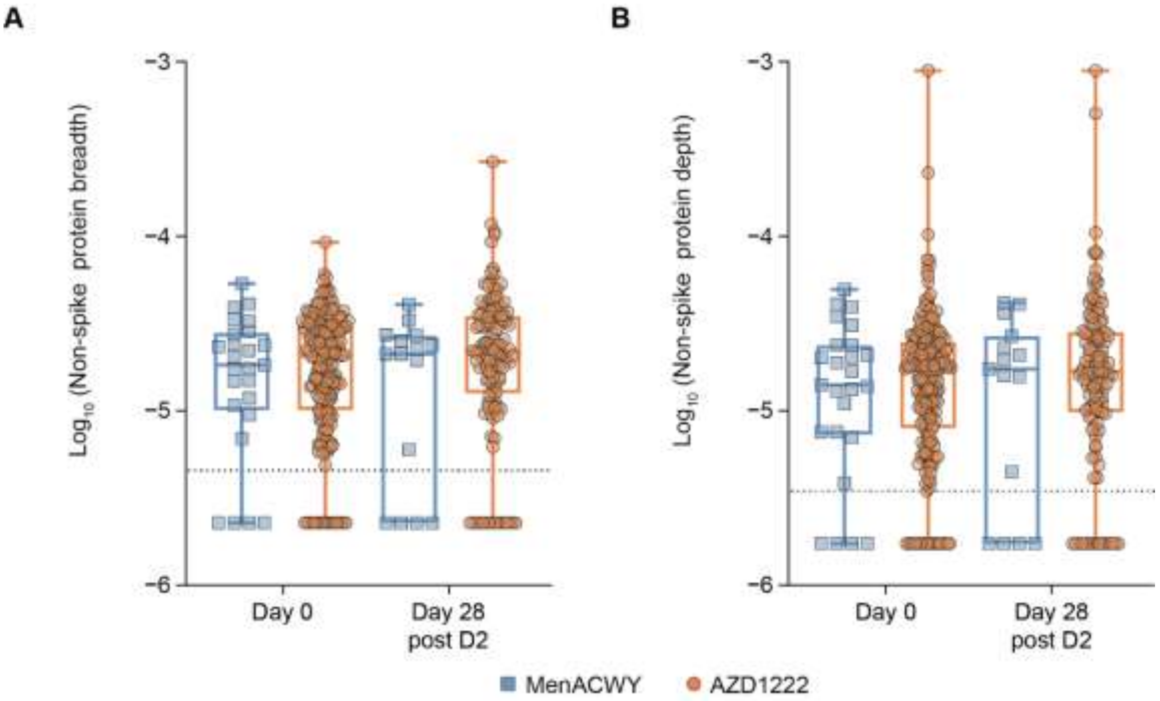

Fig. S6. Non–spike protein-specific TCR breadth and depth following vaccination with AZD1222 or MenACWY. (**A**) Non–spike protein-specific TCR breadth 28 days following second dose (D2) vaccination with MenACWY (blue) or AZD1222 (orange). (**B**) Non–spike protein-specific TCR depth following vaccination with MenACWY (blue) or AZD1222 (orange). Data are $\log_{10}$ transformed, where all zero values have been converted to half of the lowest non-zero value. Depth indicates SARS-CoV-2 associated T cells of total T cells. Breadth indicates SARS-CoV-2 associated unique TCRs of total unique TCRs. In the box and whisker plots, the horizontal line represents median, boxes represent interquartile range, whiskers extend to the min and max, and symbols represent each participant. The dotted lines represent the lower detected value. Significant differences determined by one-way ANOVA followed by Sidak's multiple comparisons tests. All comparisons are not significant unless stated as significant.

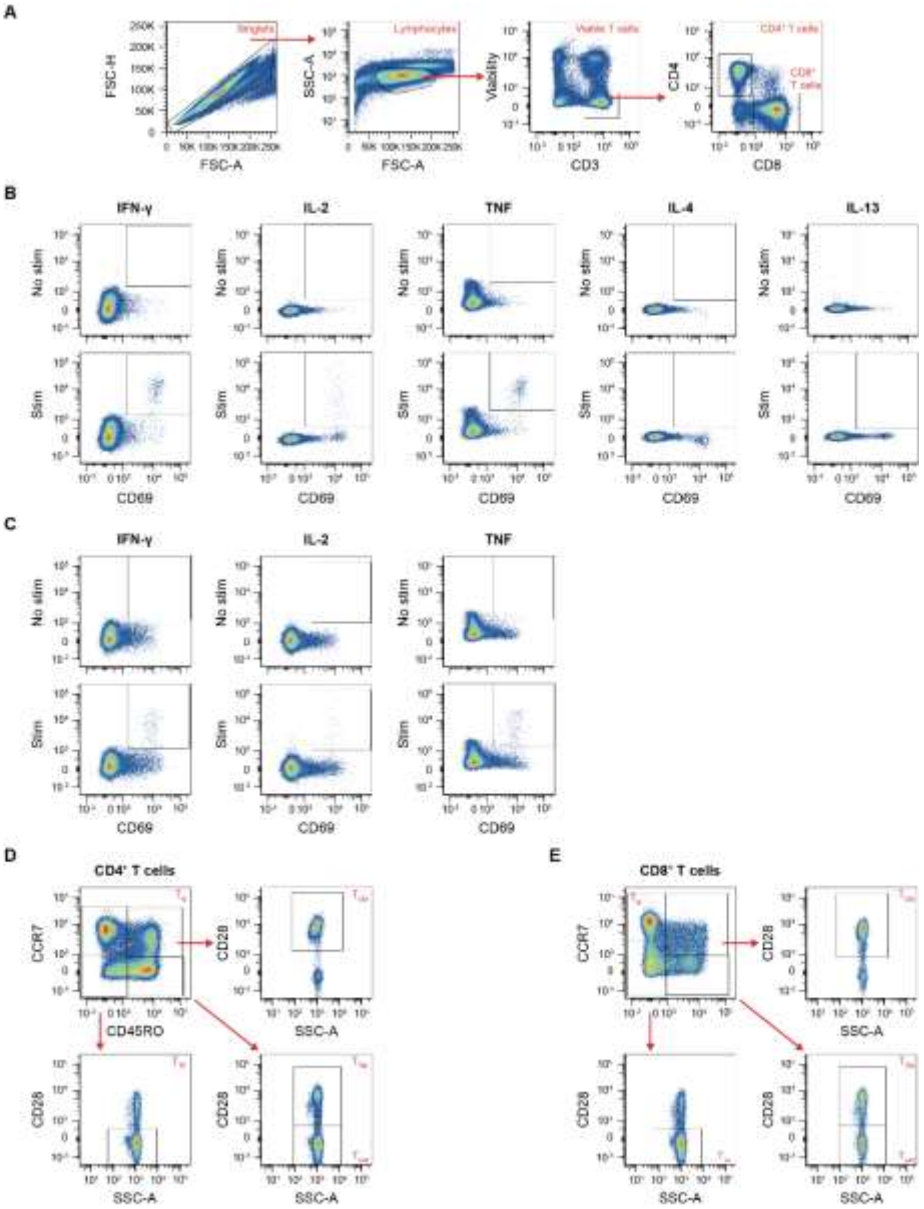

Fig. S7. Flow cytometric gating strategy. PBMCs were stained with a 13-color panel. (**A**) Lineage gating of T cells is shown. After gating on single cells, lymphocytes, and viable $CD3^+$ T cells, T cells were further subdivided into $CD4^+$ and $CD8^+$ T cells. (**B and C**) Gating to measure T cell function following no stimulation or stimulation with SARS-CoV-2 S1 peptide pool is shown. Cytokine staining is shown following gating on $CD4^+$ T cells (B) or $CD8^+$ T cells (C). (**D and E)** Memory T cell subset gating is shown for $CD4^+$ T cells (D) and $CD8^+$ T cells (E). T cell populations identified include naïve (T_N), central memory (T_{CM}), transitional memory (T_{TM}), effector memory (T_{EM}), and terminal effector (T_{TE}) cells. FSC-A, forward scatter area; FSC-H, forward scatter height; SSC-A, side scatter area.

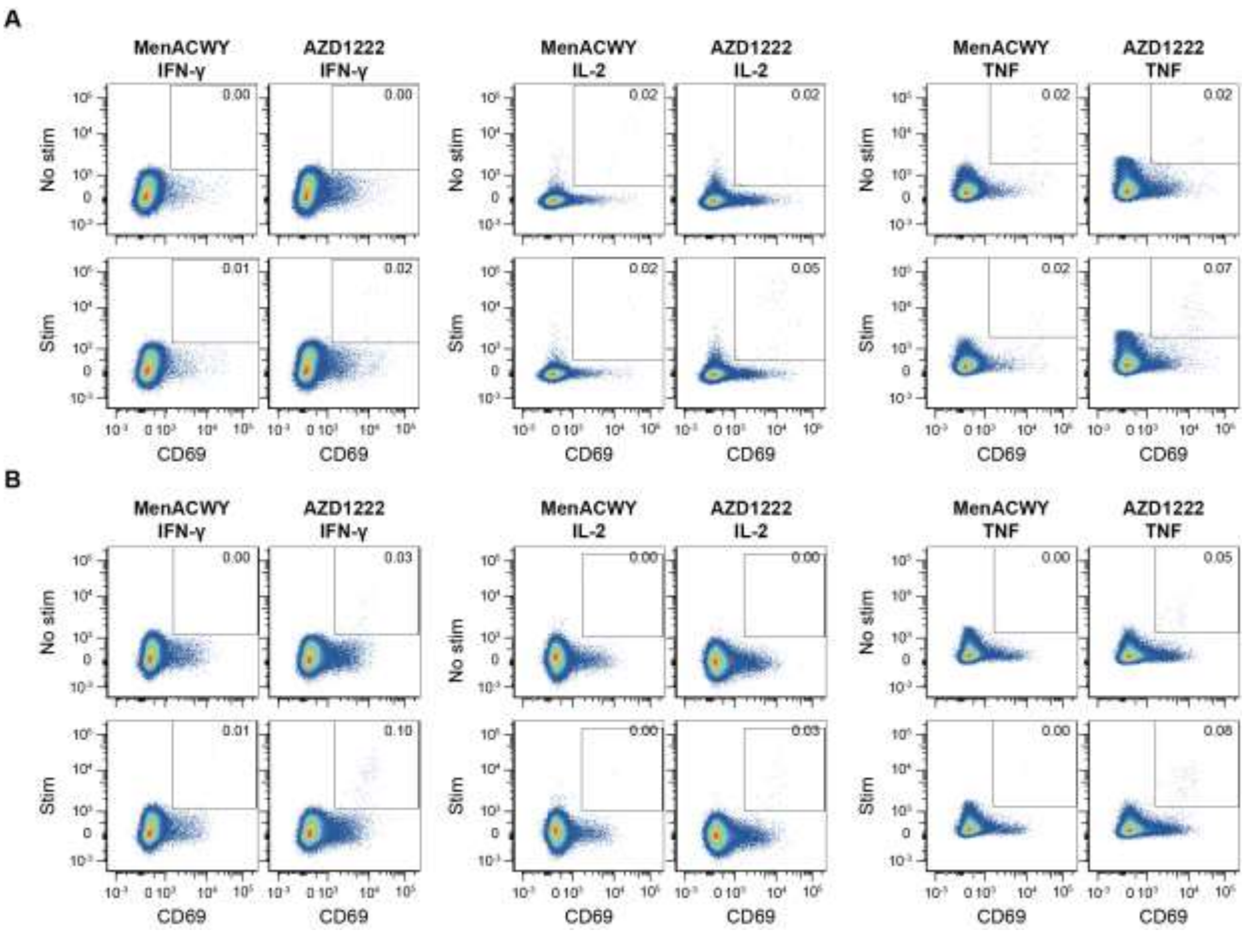

Fig. S8. Representative flow plots of T cell cytokine gating. PBMCs were stained with a 13-color panel. (**A and B**) $CD4^+$ T cells (A) and $CD8^+$ T cells (B) from MenACWY or AZD1222 vaccinated participants at Day 56 post-vaccination are shown following stimulation with dimethyl sulfoxide (DMSO, No stim) or SARS-CoV-2 S1 peptide pool (Stim). Numbers indicate the frequency of events in the indicated gate.

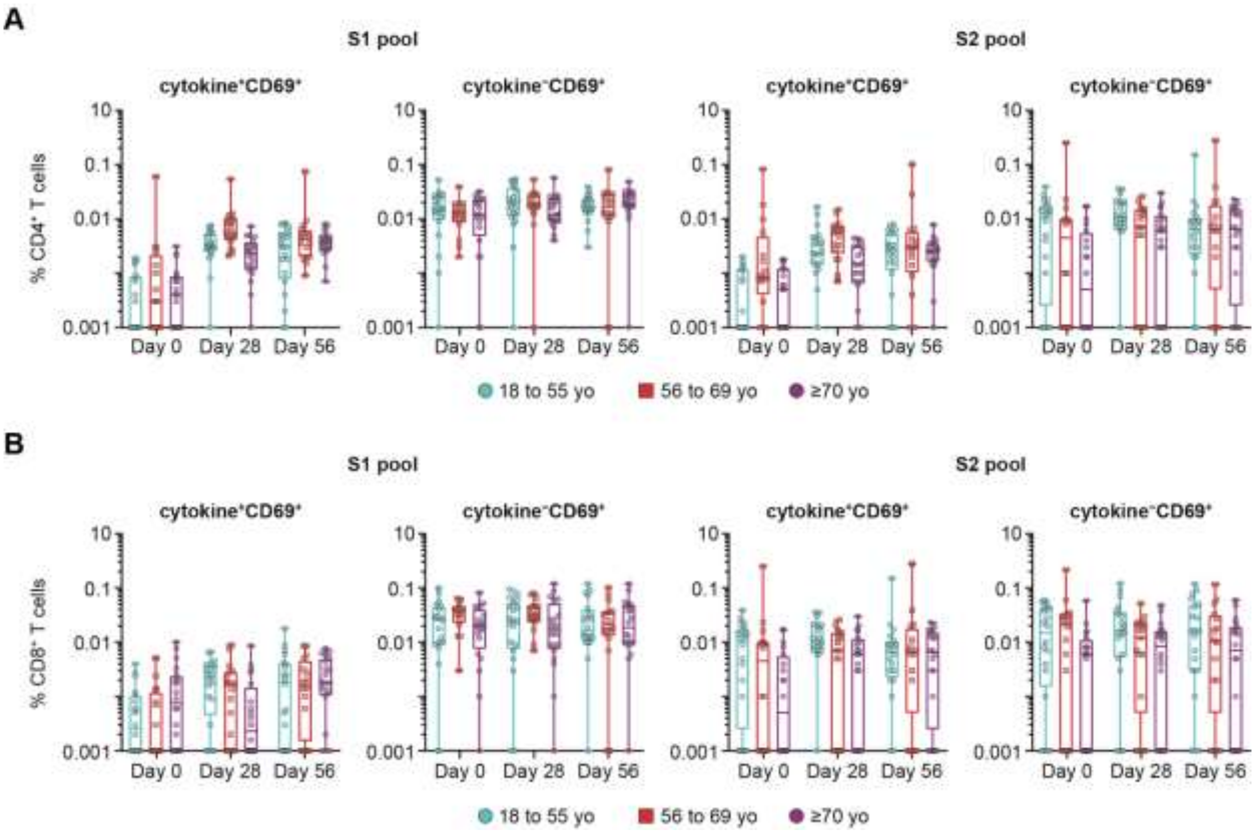

Fig. S9. T cell activation state. (**A and B**) Frequencies of spike protein-specific (cytokine^{+}) and non-specific (cytokine^{-}) CD4^{+} (A) and CD8^{+} (B) T cells expressing CD69 were measured. T cell frequencies following stimulation with S1 (left) and S2 (right) peptide pools for participants in each age group at the indicated timepoints are shown. In the box and whisker plots, the horizontal line represents median, boxes represent interquartile range, whiskers extend to the min and max, and symbols represent each participant.

Table S1. Median frequencies of spike protein-specific T cell responses. IQR, interquartile range**.**

Vaccine group	T cell type	Timepoint (Day)	Median frequency any response	IQR	Median frequency IFN-γ	IQR	Median frequency IL-2	IQR	Median frequency TNF	IQR	Median # live, $CD3^+$ events
MenACWY	$CD4^+$	0	0.02	0.00 to 0.03	0.01	0.00 to 0.03	0.00	0.00 to 0.00	0.01	0.00 to 0.02	267577
		28	0.01	0.00 to 0.02	0.00	0.00 to 0.02	0.00	0.00 to 0.01	0.00	0.00 to 0.02	296349
		56	0.02	0.01 to 0.02	0.00	0.00 to 0.04	0.00	0.00 to 0.01	0.00	0.00 to 0.04	304722
AZD1222	$CD4^+$	0	0.01	0.00 to 0.02	0.00	0.00 to 0.01	0.00	0.00 to 0.01	0.01	0.01 to 0.03	281920
		28	0.06	0.04 to 0.11	0.02	0.01 to 0.04	0.04	0.02 to 0.07	0.05	0.03 to 0.10	280084
		56	0.06	0.04 to 0.10	0.03	0.01 to 0.04	0.04	0.02 to 0.07	0.06	0.03 to 0.08	285781
COVID-19 Convalescent	$CD4^+$	-	0.13	0.12 to 0.21	0.07	0.07 to 0.09	0.09	0.07 to 0.16	0.11	0.09 to 0.16	428800
MenACWY	$CD8^+$	0	0.01	0.00 to 0.04	0.01	0.00 to 0.03	0.00	0.00 to 0.00	0.01	0.00 to 0.02	267577
		28	0.01	0.00 to 0.02	0.00	0.00 to 0.02	0.00	0.00 to 0.01	0.00	0.00 to 0.02	296349
		56	0.01	0.00 to 0.06	0.01	0.00 to 0.04	0.00	0.00 to 0.01	0.00	0.00 to 0.6	304722
AZD1222	$CD8^+$	0	0.00	0.00 to 0.02	0.00	0.00 to 0.02	0.00	0.00 to 0.00	0.00	0.00 to 0.01	281920
		28	0.02	0.00 to 0.05	0.02	0.00 to 0.05	0.01	0.00 to 0.02	0.01	0.00 to 0.03	280084
		56	0.03	0.02 to 0.07	0.03	0.01 to 0.07	0.01	0.00 to 0.03	0.02	0.00 to 0.05	285781
COVID-19 Convalescent	$CD8^+$	-	0.04	0.02 to 0.10	0.05	0.03 to 0.09	0.01	0.00 to 0.03	0.03	0.00 to 0.08	428800

Table S2. Spike protein-specific T cell response rate in AZD1222-vaccinated participants. Numbers and frequencies of AZD1222-vaccinated participants that generated a spike protein-specific $CD4^+$ and $CD8^+$ T cell response (as measured by any combination of IFN-γ, IL-2, or TNF) at Day 28 and Day 56 greater than two-fold over the corresponding response at the Day 0 timepoint are shown.

**T cell type**		**Day 28**	**Day 56**
$CD4^+$	Number of participants > two-fold response over Day 0	40	42
	Number of participants < two-fold response over Day 0	14	11
	Frequency of participants > two-fold response over Day 0	74.1%	79.2%
$CD8^+$	Number of participants > two-fold response over Day 0	24	35
	Number of participants < two-fold response over Day 0	28	16
	Frequency of participants > two-fold response over Day 0	46.2%	68.6%

Table S3. Median frequencies of spike protein-specific T cell responses with each age cohort.

Age group	T cell type	Timepoint (Day)	Median frequency any response	IQR	Median frequency IFN-γ	IQR	Median frequency IL-2	IQR	Median frequency TNF	IQR
18 to 55	$CD4^+$	0	0.00	0.00 to 0.02	0.00	0.00 to 0.00	0.00	0.00 to 0.00	0.01	0.00 to 0.02
		28	0.06	0.04 to 0.09	0.02	0.01 to 0.03	0.04	0.03 to 0.05	0.05	0.04 to 0.07
		56	0.05	0.03 to 0.10	0.03	0.01 to 0.04	0.03	0.01 to 0.07	0.05	0.03 to 0.07
56 to 60	$CD4^+$	0	0.02	0.01 to 0.05	0.01	0.00 to 0.01	0.01	0.00 to 0.03	0.02	0.01 to 0.04
		28	0.13	0.07 to 0.16	0.03	0.03 to 0.06	0.10	0.03 to 0.13	0.12	0.05 to 0.13
		56	0.08	0.04 to 0.11	0.03	0.01 to 0.06	0.05	0.03 to 0.09	0.06	0.03 to 0.10
≥70	$CD4^+$	0	0.01	0.00 to 0.02	0.00	0.00 to 0.00	0.00	0.00 to 0.01	0.01	0.00 to 0.02
		28	0.04	0.02 to 0.06	0.02	0.00 to 0.02	0.03	0.01 to 0.05	0.04	0.02 to 0.05
		56	0.06	0.04 to 0.08	0.02	0.02 to 0.03	0.04	0.02 to 0.07	0.06	0.04 to 0.07
18 to 55	$CD8^+$	0	0.00	0.00 to 0.02	0.00	0.00 to 0.01	0.00	0.00 to 0.00	0.00	0.00 to 0.01
		28	0.04	0.00 to 0.07	0.03	0.02 to 0.06	0.01	0.00 to 0.02	0.02	0.00 to 0.04
		56	0.03	0.01 to 0.12	0.03	0.01 to 0.12	0.01	0.00 to 0.04	0.04	0.01 to 0.10
56 to 60	$CD8^+$	0	0.00	0.00 to 0.03	0.01	0.00 to 0.02	0.00	0.00 to 0.00	0.00	0.00 to 0.03
		28	0.02	0.01 to 0.05	0.03	0.01 to 0.05	0.01	0.00 to 0.02	0.02	0.00 to 0.02
		56	0.03	0.02 to 0.07	0.03	0.01 to 0.05	0.02	0.01 to 0.02	0.02	0.01 to 0.04
≥70	$CD8^+$	0	0.01	0.00 to 0.03	0.00	0.00 to 0.02	0.00	0.00 to 0.00	0.00	0.00 to 0.02
		28	0.01	0.00 to 0.02	0.00	0.00 to 0.02	0.00	0.00 to 0.01	0.01	0.00 to 0.02
		56	0.03	0.02 to 0.06	0.03	0.02 to 0.07	0.01	0.00 to 0.02	0.02	0.01 to 0.04

Table S4. Spike protein-specific breadth and depth for COVID-19 convalescent participants.

	Spike protein breadth	Spike protein depth	Dosing schedule
Convalescent participant 1	7.29E-05	5.17E-05	About 4 weeks
Convalescent participant 2	5.59E-05	4.10E-05	About 4 weeks

Table S5. Top five TCRβ sequences expanded post vaccination. CDR, complementarity-determining regions; n, number of participants displaying response; TCR, T cell receptor; TRβV, T cell receptor beta variable region; TRβJ, T cell receptor beta joining region.

T cell type	CDR3 sequence	TRβV	TRβJ	Spike protein position	n
$CD4^+$	CASSEGASNQPQHF	6-1	1-5	743–801	40
$CD4^+$	CASSQGVGYTF	4-1	1-2	160–218	27
$CD8^+$	CSARGEGNYGYTF	20-X	1-2	779–790	24
$CD8^+$	CASSPDIEAFF	7-9	1-1	265–277	17
$CD4^+$	CASSRAGGSTDTQYF	7-3	2-3	372–430	15

Table S6. List of antibodies used for intracellular cytokine staining.

Detector	Fluorophore	Specificity	Clone	Dilution	Stain
B710	Brilliant Blue 700	IL-4	MP4-25D2	1:160	Intracellular
G560	Phycoerythrin	CD28	CD28.2	1:20	Surface
G610	Phycoerythrin-Texas Red-X	CD69	TP1.55.3	1:20	Intracellular
G660	Phycoerythrin-Cyanin 5.1	CD8a	RPA-T8	1:40	Surface
G780	Phycoerythrin-Cyanin 7	IFN-γ	B27	1:160	Intracellular
R660	Allophycocyanin	IL-2	MQ1-17H12	1:20	Intracellular
R780	Allophycocyanin-Hilite 7-BD	CD3	SK7	1:80	Intracellular
U395	Brilliant Ultraviolet 395	CCR7	150503	1:40	Surface
U450	Ultraviolet-Blue	Viability	-	1:500	Surface
U570	Brilliant Ultraviolet 563	TNF	Mab11	1:10	Intracellular
U785	Brilliant Ultraviolet 805	CD4	SK3	1:10	Surface
V450	Brilliant Violet 421	IL-13	JES 10-SA2	1:10	Intracellular
V785	Brilliant Violet 785	CD45RO	UCHL1	1:20	Surface
